# Analysis of the quadruple evolutionary game of ecosystem service payment empowered by farmers’ cooperatives

**DOI:** 10.1371/journal.pone.0329470

**Published:** 2025-09-04

**Authors:** Huimin Shao, Wenjuan Zhang, Liangyan Lu, Walton Wider, Yihang Guo

**Affiliations:** 1 School of Management, Yunnan Normal University, Kunming, China,; 2 School of Economics, Yunnan Normal University, Kunming, China,; 3 Department of Accounting and Finance, Yunnan University of Business Administration, Kunming, China,; 4 School of Business and Communication, InTI International University, Nilai, Negeri Sembilan, Malaysia; University of Vermont, UNITED STATES OF AMERICA

## Abstract

Payments for Ecosystem Services (PES) are essential for ecosystem restoration and promoting sustainable economic development. Farmer cooperatives serve as key intermediaries in implementing PES. This study constructs a game model involving four stakeholders—local government, enterprises, cooperatives, and cooperative members—while considering their bounded rationality. Numerical simulations using Matlab are conducted to test the stability and effectiveness of equilibrium strategies among these stakeholders. The results show that no matter which direction the system evolves, the strategy of farmers ‘ cooperatives is to produce ecological products, that is, to effectively promote the realization of the value of ecological products, farmers ‘ cooperatives need to actively participate in the production of ecological products. The payment model of ecosystem services can not only provide practical basis and motivation for the payment of ecosystem services, promote the optimization and improvement of relevant mechanisms, but also enhance the market competitiveness of farmers ‘cooperatives and enhance their brand value.

## 1. Introduction

In the context of global economic integration and sustainable development goals, the protection and sustainable use of ecosystem services has become the focus of attention of the international community. Ecosystem services not only provide basic living conditions for human beings, such as water, air and food, but also play an important role in regulating climate, maintaining biodiversity and promoting socio-economic development(Zhang Zhenzhen et al.,2022) [1]. With the acceleration of population growth and urbanization, the ecosystem is facing unprecedented pressure, resulting in the deterioration of the ecological environment, which in turn affects the quality of human life. Therefore, how to effectively protect and utilize ecosystem services has become a major issue to be solved globally.

**Payments for Ecosystem Services (PES)** reflect the comprehensive economic, ecological, and social value of ecological products through government-led initiatives, social participation, and market-based operations (Wang Jinnan & Wang Xiahui, 2020) [2]. PES can effectively reduce environmental pollution and restore ecosystems. Rural areas, rich in ecological products, are ideal for exploring PES mechanisms, which contribute to both ecological revitalization and industrial rejuvenation, while promoting green, high-quality agricultural development (Ouyang Zhenyi & Xie Hualin, 2023) [3]. This, in turn, supports the development of a low-carbon economy. However, government-led PES initiatives in rural areas remain insufficient, often characterized by a “government acts, farmers observe” phenomenon, where farmers’ interests are not fully addressed(Fan Shengyue et al., 2022) [4].

Farmer cooperatives, as mutual assistance economic organizations formed by vulnerable groups, are primarily aimed at maximizing the benefits of their members (Xu Xuchu & Wu Bin, 2018) [5]. This makes cooperatives ideal intermediaries for transforming PES into tangible benefits. In the PES process, farmer cooperatives play an indispensable role by extending the industrial and value chains of ecological products, increasing revenues and market shares from ecological product development, safeguarding the interests of cooperative members involved in ecological production, and contributing to rural ecological governance.

The purpose of this study is to explore the role and evolution mechanism of farmers ‘ cooperatives in the payment of ecosystem services from the perspective of evolutionary game theory. Evolutionary game theory provides a powerful tool for analyzing how individuals choose strategies in a dynamic environment and can reveal the complex relationship between cooperation and competition. In the payment mechanism of ecosystem services, farmers ‘ cooperatives, as participants, are not only affected by internal members, but also restricted by external market environment and policy framework(Fan Shengyue et al., 2022) [6]. By constructing an evolutionary game model, the study will focus on analyzing the behavior choices of farmers ‘ cooperatives and their impact on ecosystem services under different ecological compensation mechanisms.

At present, the international research on ecosystem services is gradually developing towards diversification and systematization, especially in the design and implementation of payment ecosystem services (PES) mechanism(Liu Jiemei et al.,2024) [7]. However, there is still a lack of in-depth theoretical discussion and empirical support on the role of farmers ‘cooperatives in this mechanism, especially the interaction between its internal governance structure and the external market environment. Therefore, this study not only fills the theoretical gap in this field, but also provides new perspectives and ideas for policy makers, academia and practitioners.

The innovation of this study is that, first of all, the evolutionary game theory is introduced into the study of farmers ‘ cooperatives and ecosystem service payment, revealing the game relationship between the members of the cooperative and its impact on the provision of ecological services. Secondly, the study will explore how farmers ‘ cooperatives can achieve the dual benefits of ecology and economy by adjusting the internal governance structure and external cooperation relationship under different ecological compensation mechanisms. Finally, through case analysis and model simulation, the research will provide empirical basis and policy recommendations for the sustainable development of farmers ‘ cooperatives.

## 2. Literature review

**Farmer cooperatives** have been recognized as playing a critical role in implementing Payments for Ecosystem Services (PES), a point that has garnered widespread attention in academic research. First, the cooperative model has been identified as an effective mechanism for promoting farmers’ direct participation in the production and sale of ecological products. It has been found that cooperatives, through collective action, help their members overcome barriers related to market information and capital accumulation, thereby enhancing the market competitiveness of ecological products (Zhao Xiaofeng & Xing Chengju, 2016; Gao Yuan & Ma Jiu Jie, 2024) [8,9]. This model has proven particularly suitable for products with high ecological value and environmental sensitivity, while also accommodating small-scale farmers by creating a community that integrates “rights, responsibilities, and benefits” (Bosselmann & Lund, 2013) [10]. Second, regarding the functional expansion of cooperatives in realizing the value of ecological products, studies have suggested that cooperatives can enhance the perceived value and market acceptance of these products through innovative management and marketing strategies (Zhang Ying & Yuan Peng, 2023) [11]. In practice, Zhejiang has established and operated “Two Mountains Cooperatives,” providing a new platform for managing ecological resource assets and implementing PES (Jin Chunhua et al., 2023) [12]. Case studies focusing on specific agricultural practices, such as rice cultivation and traditional Chinese medicinal herb farming, have analyzed how cooperatives implement PES and have proposed corresponding optimization strategies (Xie Jianwei, 2023; Dou Hanyang et al., 2024) [13,14]. These studies have not only deepened the understanding of how cooperatives promote PES but have also provided strategies to address challenges posed by market and ecological pressures.

**Evolutionary game theory** has been employed as a framework for studying how interactions among individuals in a population evolve over time(Pan Feng et al., 2022) [15]. By integrating game theory with dynamic process analysis, evolutionary game theory has helped researchers understand the strategic and behavioral dynamics within complex systems. In economics, evolutionary game models have been used to explain these dynamics, and current models have primarily included two-party(Zhang Jinquan et al., 2023) [16], three-party, and four-party games(Cui Ning et al., 2023) [17], with three-party models being more widely applied. As PES has gained increasing attention, researchers have constructed various game models to explore PES for marine ecological products, forest products, and ecological agricultural products. For example, a three-party evolutionary game model involving enterprises, government, and the public has been developed to provide a theoretical basis for policy development aimed at promoting marine ecological civilization and high-quality economic growth (Shi Yaping & Zhu Qinglin, 2023) [18]. Similarly, a four-party model, based on the assumption of bounded rationality among stakeholders, has incorporated local governments, banks, forestry enterprises, and consumers to explore stable equilibrium strategies for realizing the value of forest ecological products (Zhang Jianing & Hu Xiaofei, 2023) [19]. Research on PES mechanisms, largely based on the perspective of evolutionary game theory, has generally been divided into two main areas: the “supply-demand” aspect, which examines the relationships between suppliers, consumers, government, and markets (Zhang Hongrui & Liu Xin, 2021) [20], and the “subsidy” aspect, which investigates the equilibrium outcomes expected after the introduction of additional subsidy policies (Qi Xiaoxing et al., 2023) [21].

However, although the existing research has made some progress in many aspects, there are still some shortcomings. First of all, the research on the specific mechanism of cooperatives in the realization of the value of ecological products is still insufficient, especially in the comparative analysis between different types of ecological products(Xie Hualin & Li Zhiyuan, 2023) [22]. Secondly, the existing literature is more concerned about the three parties involved in the main body, including the government, enterprises and consumers, etc., and contains local governments, enterprises, cooperatives, members of the four parties involved in the main body of the study is less. In addition, although existing research has focused on the relationship between supply and demand and subsidy policies, the discussion on the dynamic interaction mechanism between the internal governance structure of cooperatives and changes in the external environment is still weak.

## 3. Research methodology

### 3.1 Evolutionary game model analysis

#### 3.1.1 Model assumptions.

In constructing the game model based on relevant policies and existing research, the following assumptions are made as the foundation for analysis:

Assumption 1: Actors and Strategies. The local government’s strategies include “incentive” and “inaction,” where the probability of adopting an incentive strategy is denoted as x, and the probability of adopting an inaction strategy is 1 − x. The enterprise’s strategies include “ecological product management” and “traditional product management,” with the respective probabilities y and 1 − y,the enterprise here refers to the profit-making organization engaged in the processing and sales of agricultural products. The cooperative’s strategies include “ecological product production” and “traditional product production,” with probabilities z and 1 − z, respectively. Farmers’ strategies involve “joining the cooperative” and “not joining the cooperative,” where the probability of joining is m and the probability of not joining is 1 − m. Therefore, x,y,z,m∈[0,1].

Among these four actors, the actors are farmers ‘ cooperatives. Farmers ‘ cooperatives can integrate members ‘ land, labor, capital and other resources, improve the degree of organization of agricultural production, and better adapt to the scale and standardization requirements of ecological product production. It can also guide members to adopt eco-friendly production methods, promote the production of green, organic and geographically labeled agricultural products, and promote the sustainable development of agriculture. In this process, the government has played a guiding and supporting role. It encourages farmers ‘ cooperatives to participate in projects that realize the value of ecological products by formulating policies, providing financial subsidies, and tax incentives. Farmers ‘ cooperatives and enterprises can cooperate through the mode of ‘ leading enterprises + cooperatives + farmers ‘ or ‘ cooperatives + enterprises + farmers ‘. In this mode, enterprises can take advantage of the organizational advantages of cooperatives to obtain a stable supply of raw materials and reduce transaction costs; cooperatives can use the capital, technology and market channels of enterprises to enhance the added value and market competitiveness of ecological products.

Assumption 2: Costs and Subsidies. When the local government adopts an incentive policy, it engages in ecological product certification, which requires corresponding policy measures and dedicated personnel for implementation. The cost incurred by the government for this process is C_1_. During the certification process, the government collects certification fees A from producers. Enterprises incur a cost C_2_ for managing ecological products and C_3_ for managing traditional products, where C_2_ > C_3_. Cooperatives face costs of C_4_ for ecological product production and C_5_ for traditional product production, with C_4_ > C_5_. When both enterprises and cooperatives engage in ecological product production and management, they can collaborate to extend the industrial chain of ecological products and promote eco-tourism projects. In this process, enterprises incur an additional cost C_6_, and cooperatives incur C_7_. To encourage enterprises to engage in ecological product management and cooperatives to grow ecological products, the government provides technical support S_1_ to enterprises and financial assistance S_2_ to cooperatives.

Assumption 3: Market Gains and Losses. If enterprises engage in ecological product management, they can earn sales revenue E_1_, whereas traditional product management yields revenue E_2_. Cooperatives earn revenue E_3_ from ecological product production and E_4_ from traditional product production. Enterprises also gain revenue E_5_ from eco-tourism projects, while cooperatives earn revenue E_6_. Through brand development and marketing, cooperatives can enhance the recognition and competitiveness of ecological products, allowing them to charge a premium R when selling such products. However, brand promotion and marketing incur an additional cost C_a_. To promote PES, cooperatives invite experts and technicians to provide green agricultural training and guidance to members, reducing pesticide use and pollution, thus supporting the cooperative’s green transition. The cost of this process is C_b_. Cooperatives can also integrate dispersed rural ecological resources such as arable land, forests, and water bodies through unified planning and management, improving resource utilization efficiency. This leads to economies of scale E_m_, increasing producers’ earnings. When cooperatives engage in ecological product production, members participating in this production receive benefits U_1_; when cooperatives produce traditional products, members receive benefits U_2_.The farmers ‘ cooperative is a mutual economic organization, and the members refer to the farmers who join the farmers ‘ cooperative. The income obtained by the cooperative will be returned according to the proportion of the members and the transaction volume of the society. The reason why members have different strategies from cooperatives is that members who join cooperatives have different individual characteristics, and their educational level or production scale may affect their willingness to produce; in addition, if the benefit distribution mechanism of the cooperative is unreasonable, such as the proportion of dividends is too low or the distribution is not transparent, members may also be resistant to the production objectives of the cooperative. If members’ production preferences do not align with the cooperative’s production, they receive reduced benefits of rU_2_ (0 ≤ r ≤ 1). Members involved in ecological product production can also acquire skill enhancements B through training provided by the cooperative, and when cooperatives implement eco-tourism projects, members can earn additional income F.

Assumption 4: Potential Gains and Losses. When the government certifies ecological products, it earns a social reputation benefit E_a_. Enterprises or cooperatives involved in ecological product production gain environmental benefits E_b_, while the government incurs a social reputation loss E_c_ if it chooses inaction. Cooperatives can serve as role models, demonstrating successful PES experiences and models that encourage more farmers to participate in ecological protection, generating social benefits E_n_. Enterprises engaged in ecological product management promote the commercialization of these products, fulfilling corporate social responsibility and thereby gaining social benefits Ep.

#### 3.1.2 Model construction.

Based on the above assumptions and parameter settings, the mixed-strategy game matrix for the local government, enterprises, cooperatives, and cooperative members is shown in Table 1.

**Table 1:** Mixed-strategy game matrix for the four actors: local government, enterprises, cooperatives, and cooperative members.

		Cooperative Ecological Product Production		Cooperative Traditional Product Production	
		Members Participate in Ecological Product Production	Members Participate in Traditional Product Production	Members Participate in Ecological Product Production	Members Participate in Traditional Product Production
**Local Government Incentives** (Incentive)	Enterprise Ecological Product Management	a_1_:-C_1_-S_1_-S_2_ + 2A + E_a_ + E_b_ b_1_:-C_2_-C_6_-A + S_1_ + E_1_ + E_p_ + E_5_ c_1_:-C_4_-C_7_-A + S_2_ + E_3_ + R + E_6_-C_a_-C_b_ + E_m_ + E_n_ d_1_:U_1_ + B + F	a_2_:-C_1_-S_1_-S_2_ + 2A + E_a_ + E_b_ b_2_:-C_2_-C_6_-A + S_1_ + E_1_ + E_p_ + E_5_ c_2_:-C_4_-C_7_-A + S_2_ + E_3_ + R + E_6_-C_a_-C_b_ + E_m_ + E_n_ d_2_:rU_2_	a_3_:-C_1_-S_1_ + A + E_a_ + E_b_ b_3_:-C_2_-A + S_1_ + E_1_ + E_p_ c_3_:-C_5_ + E_4_ d_3_:rU_2_	a_4_:-C_1_-S_1_ + A + E_a_ + E_b_ b_4_:-C_2_-A + S_1_ + E_1_ + E_p_ c_4_:-C_5_ + E_4_ d_4_:U_2_
	Enterprise Traditional Product Management	a_5_:-C_1_-S_2_ + A + E_a_ + E_b_ b_5_:-C_3_ + E_2_ c_5_:-C_4_-A + S_2_ + E_3_ + R-C_a_-C_b_ + E_m_ + E_n_ d_5_:U_1_ + B	a_6_:-C_1_-S_2_ + A + E_a_ + E_b_ b_6_:-C_3_ + E_2_ c_6_:-C_4_-A + S_2_ + E_3_ + R-C_a_-C_b_ + E_m_ + E_n_ d_6_:rU_2_	a_7_:-C_1_ + E_a_ b_7_:-C_3_ + E_2_ c_7_:-C_5_ + E_4_ D_7_:rU_2_	a_8_:-C_1_ + E_a_ b_8_:-C_3_ + E_2_ c_8_:-C_5_ + E_4_ d_8_:U_2_
**Local Government Inaction** (No Action)	Enterprise Ecological Product Management	a_9_:E_b_-E_c_ b_9_:-C_2_-C_6_ + E_1_ + E_p_ + E_5_ c_9_:-C_4_-C_7_ + E_3_ + R + E_6_-C_a_-C_b_ + E_m_ + E_n_ d_9_:U_1_ + B + F	a_10_:E_b_-E_c_ b_10_:-C_2_-C_6_ + E_1_ + E_p_ + E_5_ c_10_:-C_4_-C_7_ + E_3_ + R + E_6_-C_a_-C_b_ + E_m_ + E_n_ d_10_:rU_2_	a_11_:E_b_-E_c_ b_11_:-C_2_ + E_1_ + E_p_ c_11_:-C_5_ + E_4_ d_11_:rU_2_	a_12_:E_b_-E_c_ b_12_:-C_2_ + E_1_ + E_p_ c_12_:-C_5_ + E_4_ d_12_:U_2_
	Enterprise Traditional Product Management	a_13_:E_b_-E_c_ b_13_:-C_3_ + E_2_ c_13_:-C_4_ + E_3_ + R-C_a_-C_b_ + E_m_ + E_n_ d_13_:U_1_ + B	a_14_:E_b_-E_c_ b_14_:-C_3_ + E_2_ c_14_:-C_4_ + E_3_ + R-C_a_-C_b_ + E_m_ + E_n_ d_14_:rU_2_	a_15_:-E_c_ b_15_:-C_3_ + E_2_ c_15_:-C_5_ + E_4_ d_15_:rU_2_	a_16_:-E_c_ c_16_:-C_3_ + E_2_ c_16_:-C_5_ + E_4_ d_16_:U_2_

### 3.2 Strategy stability analysis of game participants

#### 3.2.1 Strategy stability analysis of the local government.

When analyzing the strategy choices of the local government, the expected payoff for the “certification” strategy is denoted as E_11_, while the expected payoff for the “inaction” strategy is denoted as E_12_.


$$\begin{array}{c} E_{11}=yzma_{1}+yz(1-m)a_{2}+ym(1-z)a_{3}+y(1-z)(1-m)a_{4}+zm(1-y)a_{5}+z(1-y)(1-m)a_{6}+\\ m(1-y)(1-z)a_{7}+(1-y)(1-z)(1-m)a_{8}\\ E_{12}=yzma_{9}+yz(1-m)a_{10}+ym(1-z)a_{11}+y(1-z)(1-m)a_{12}+zm(1-y)a_{13}+z(1-y)(1-m)a_{14}\\ +m(1-y)(1-z)a_{15}+(1-y)(1-z)(1-m)a_{16} \end{array}$$

The average expected payoff is: ${\overset{―}{E}}_{1}=xE_{11}+(1-x)E_{12}$

The replicator dynamic equation is: $F(x)=x(E_{11}-{\overset{―}{E}}_{1})=x(1-x)(E_{11}-E_{12})=x(1-x)\phi \left(y,z,m\right)$


$$\phi (y,z,m)=-C_{1}-zS_{2}+zA-yS_{1}+yA+Ea+Ec$$

The first derivative of this function is: F′(x)=(1−2x)ϕ(y,z,m)

As can be seen from the above formula, the main factors affecting the local government strategy are the probability of decision-making by enterprises and cooperatives, the cost of ecological product certification, the cost of ecological product certification by local governments, the technical support provided by local governments to enterprises, the financial support provided to cooperatives, social reputation benefits and social reputation loss.Therefore, local governments should establish a unified certification standard and evaluation system for ecological products in coordination with relevant departments, so as to avoid repeated certification and inconsistent standards. This can not only reduce the certification cost of enterprises and cooperatives, but also improve the market recognition of ecological products. It can also achieve information query, quality traceability, and responsibility traceability by establishing an ecological product quality traceability mechanism, thereby increasing social reputation benefits. In order to ensure that the local government strategy is stable, certain conditions should be met: F(x)=0 and F’(x)<0.

Let $z_{0}=(C_{1}+yS_{1}-yA-Ea-Ec)/(A-S_{2})$, when **z = z**_**0**_, F (x) 0, F(x)=0, meaning that x∈[0,1]represents stable points. When **z ≠ z**_**0**_, from F(x)=0, it can be concluded that both the “incentive” strategy (x = 1) and the “inaction” strategy (x = 0) are stable strategies for the local government.

Proposition 1: When “inaction” is the stable strategy for the local government, the condition 0 < z < z_0_ must be satisfied. Conversely, when “incentive” is the stable strategy for the local government, the condition z_0_ < z < 1must be satisfied. When z = z_0,_ the stability of the strategy cannot be determined.

**Fig 1:**
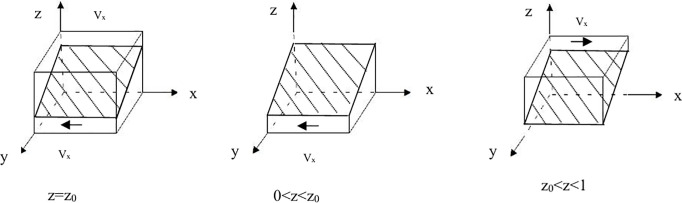
Phase diagram of the local government’s strategy. **(A)**The Phase Diagram of the Local Government’s Strategy when z = z_0._ **(B)**The Phase Diagram of the Local Government’s Strategy when 0 < z < z_0._ **(C)**The Phase Diagram of the Local Government’s Strategy when z_0_ < z < 1.

Evidence that: $\varphi (y,z,m)/z=A-S_{2}>0$, namely, φ(y, z, m) is an increasing function of z, when 当$0<z<z_{0},\phi (y,z,m)<0$, by $F(x){|}_{x=0}=0,F\prime (x){|}_{x=0}<0$, then x = 0 has stability. When $z_{0}<z<1,\phi (y,z,m)>0$, from $F(x){|}_{x=1}=0,\;F\prime (x){|}_{x=1}<0$, then x = 1 is stable. Based on the above analysis, the phase diagram of the local government strategy can be obtained.See Fig 1A–C.

Let V_x0_ represent the probability of the “inaction” strategy adopted by local governments, and V_x1_ represent the probability of the “certification” strategy by local governments.


$$\begin{array}{c} V_{x0}=\int_{0}^{1}\int_{0}^{1}((C_{1}+yS_{1}-yA-Ea-Ec)/(A-S_{2}))dxdy=(C_{1}-\frac{1}{2}A+\frac{1}{2}S_{1}-Ea-Ec)/(A-S2)\\ V_{x1}=1-V_{x0}=1-(C1-\frac{1}{2}A+\frac{1}{2}S_{1}-Ea-Ec)/(A-S_{2}) \end{array}$$

Corollary 1: If the cost of ecological product certification, social reputation gains and social reputation losses increase, in this case, the increase in the cost of ecological product certification will increase the payment received by local governments. When the social reputation gains or losses increase, it will prompt local governments to promote the relevant policies of ecological products. At this time, local governments will choose incentive policies; when the cost of local government certification of ecological products, the technical support given to enterprises and the financial support given to cooperatives are reduced, it means that local government expenditures will be reduced, and local governments will choose incentive policies.

Evidence that:


$$\partial V_{x1}/\partial A>0,\;\partial V_{x1}/\partial Ea>0,\;\partial V_{x1}/\partial Ec>0,\;\partial V_{x1}/\partial C_{1}<0,\;\partial V_{x1}/\partial S_{1}<0,\;\partial V_{x1}/\partial S_{2}<0.$$

#### 3.2.2 Stability analysis of enterprise strategy.

The expected benefits of the “ecological product management” and “traditional product management” are E_21_ and E_22_;


$$\begin{array}{c} E_{21}=xzmb_{1}+xz(1-m)b_{2}+xm(1-z)b_{3}+x(1-z)(1-m)b_{4}+zm(1-x)b_{9}+z(1-x)(1-m)b_{10}+\\ m(1-x)(1-z)b_{11}+(1-x)(1-z)(1-m)b_{12}\\ E_{22}=xzmb_{5}+xz(1-m)b_{6}+xm(1-z)b_{7}+x(1-z)(1-m)b_{8}+zm(1-x)b_{13}+z(1-x)(1-m)b_{14}+\\ m(1-x)(1-z)b_{15}+(1-x)(1-z)(1-m)b_{16} \end{array}$$

The average expected benefit is: ${\overset{―}{E}}_{2}=yE_{21}+(1-y)E_{22}$

The dynamic equation is expressed as:


$$F(y)=y(E_{21}-\overset{―}{E}2)=y(1-y)(E_{21}-E_{22})=y(1-y)\theta (x,z,m)$$

$$\theta (x,z,m)=zE_{5}-zC_{6}+xS_{1}-xA-C_{2}+C_{3}+E_{1}+Ep-E_{2}$$

The first derivative of this function is: F′(y)=(1−2y)θ(x,z,m)

From the above equation, it can be deduced that the main factors influencing the enterprise’s strategy are the probability of decisions made by other participants, the technical support provided by the local government to the enterprise, the costs of ecological product management and traditional product management, the sales revenue from both ecological and traditional product management, the costs and benefits of eco-tourism projects, as well as the social benefits brought by product commercialization.Local governments should provide enterprises with financial subsidies for ecological product certification, production and technological transformation, and give tax relief to enterprises engaged in ecological product management to reduce their operating costs. It can also integrate tourism resources in the region, carry out unified market development and marketing, attract more tourists, and increase the passenger flow and income of ecotourism projects. For the enterprise’s ecological product management strategy to be in a stable state, the following conditions must be satisfied: F(y)=0 and F’=(y)<0.

Let $z_{0}=(xA-xS_{1}+C_{2}+E_{2}-C_{3}-E_{1}-Ep)/(E_{5}-C_{6})$, When z = z_0_, F(y)0, indicating that any y∈[0,1] can be considered a stable point. When zz_0_, from F(y)0, it follows that both “ecological product operation (y=1)” and “traditional product operation (y=0)” are stable strategies.

**Fig 2:**
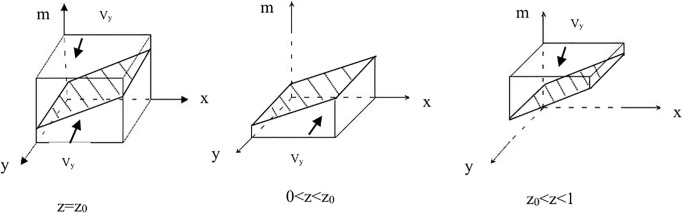
Phase diagram of the enterprise strategy. **(A)**The phase diagram of the enterprise strategy when z = z_0._ **(B)**The phase diagram of the enterprise strategy when 0 < z < z_0._ **(C)**The phase diagram of the enterprise strategy when z_0_ < z < 1.

**Proposition 2**: When a firm tends toward “traditional product operation” as a stable strategy, the condition 0 < z < z_0_ must be met. Conversely, if a firm opts for “ecological product operation” as a stable strategy, the requirement is z_0_ < z < 1. When z = z_0_, it is impossible to definitively determine the stable strategy of the firm.See Fig 2A–C.

The probabilities for a company adopting different strategic approaches can be represented as follows: V_y0_ indicates the likelihood of the company employing a “traditional product management” strategy, while V_y1_ represents the probability of pursuing an “ecological product management” strategy. Based on the calculations, these probabilities provide a quantitative foundation for evaluating strategic options.


$$\begin{array}{c} V_{y0}=\int_{0}^{1}\int_{0}^{1}((xA-xS1+C2+E2-C3-E1-Ep)/(E_{5}-C_{6}))dxdy=(\frac{1}{2}A-\frac{1}{2}S_{1}+C_{2}+E_{2}-C_{3}-E1-Ep)/(E_{5}-C_{6})\\ V_{y1}=1-V_{y0}=1-(A-S_{1}+C_{2}+E_{2}-C_{3}-E_{1}-Ep)/(E_{5}-C_{6}) \end{array}$$

Inferences 2: The enterprise chooses the strategy of the ecological product operation when the sales income, the cost of the traditional product operation, the sales support of the local government, the ecological product operation, the sales income of the traditional product operation and the ecological product certification cost are reduced.

Prove: obtain the probability of


$$\begin{array}{c} \partial V_{y1}/\partial E_{1}>0,\partial V_{y1}/\partial C_{3}>0,\partial V_{y1}/\partial S_{1}>0,\partial V_{y1}/\partial E_{5}>0,\partial V_{y1}/\partial Ep>0,\partial V_{y1}/\partial C_{2}<0,\\ \partial V_{y1}/\partial C_{6}<0,\partial V_{y1}/\partial E_{2}<0,\partial V_{y1}/\partial A<0. \end{array}$$

#### 3.2.3 Stability analysis of cooperative strategy.

The expected benefits of the cooperative in “ecological production” and “traditional production” are E_31_ and E_32_:


$$\begin{array}{c} E_{31}=xymc_{1}+xy(1-m)c_{2}+xm(1-y)c_{5}+x(1-y)(1-m)c_{6}+ym(1-x)c_{9}+y(1-x)(1-m)c_{10}+\\ m(1-x)(1-y)c_{13}+(1-x)(1-y)(1-m)c_{14}\\ E_{32}=xymc_{3}+xy(1-m)c_{4}+xm(1-y)c_{7}+x(1-y)(1-m)c_{8}+ym(1-x)c_{11}+y(1-x)(1-m)c_{12}+\\ m(1-x)(1-y)c_{15}+(1-x)(1-y)(1-m)c_{16} \end{array}$$

The average expected benefit is: ${\overset{―}{E}}_{3}=zE_{31}+(1-z)E_{32}$

The dynamic equation is expressed as:


$$F(z)=z(E_{31}-{\overset{―}{E}}_{3})=z(1-z)(E_{31}-E_{32})=z(1-z)\rho \left(x,y,m\right)$$

$$\rho (x,y,m)=yE_{6}-yC_{7}+xS_{2}-xA-C_{4}+E_{3}+R-Ca-Cb+Em+En+C_{5}-E_{4}$$

The first derivative of this function is: F′(z)=(1−2z)ρ(x,y,m)

From the above equation, it can be seen that the main factors influencing the cooperative’s strategic decisions include the probability of decisions made by other entities, the costs associated with ecological product certification, financial support from local governments, product promotion premiums, the costs and benefits of ecological and traditional product production, the costs and returns of ecological tourism projects, expenses related to brand promotion and marketing, costs of technical training, economies of scale in production, and social benefits. For the cooperative’s strategy of ecological product production to remain stable, certain conditions must be met: F(z)=0且F’(z)<0.

Order $y_{0}=(-xS_{2}+xA+C_{4}-E_{3}-R+Ca+Cb-Em-En-C_{5}+E_{4})/(E_{6}-C_{7})$, when y = y_0_, F(z)0, so z[0,1]is a stability point; when yy_0_, F(z)0 can launch the cooperative for “ecological product production” (z = 1) and “traditional product management” (z = 0).

**Fig 3:**
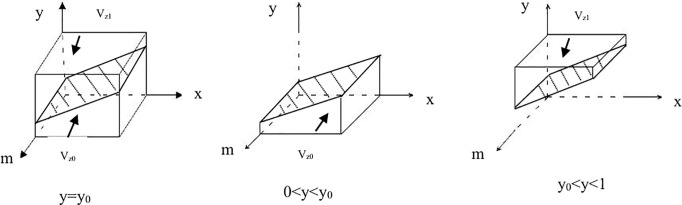
Phase diagram of cooperative strategies. **(A)** The phase Diagram of Cooperative Strategieswhen y = y_0._ **(B)**The phase Diagram of Cooperative Strategieswhen 0 < y < y_0._ **(C)**The phase Diagram of Cooperative Strategieswhen y_0_ < y < 1.

Proposition 3: When the cooperative’s stable strategy is “traditional product production,” the condition 0 < y < y_0_ must be satisfied. When the stable strategy is “ecological product production,” the condition y_0_ < y < 1; must hold. If y = y_0_, the stability of the strategy cannot be determined.Based on the above analysis, the phase diagram of the cooperative strategy can be obtained.See Fig 3A–C.

V_z0_ and V_z1_ represent the probabilities of the cooperative adopting the “ecological product production” strategy and the “traditional product production” strategy, respectively. The calculated as follows:


$$\begin{array}{c} V_{z0}=\int_{0}^{1}\int_{0}^{1}((-xS_{2}+xA+C_{4}-E_{3}-R+Ca+Cb-Em-En-C_{5}+E_{4})/(E_{6}-C_{7}))dxdm=(\frac{1}{2}A-\frac{1}{2}S_{2}+C_{4}-E_{3}-R\\ +Ca+Cb-Em-En-C_{5}+E_{4})/(E_{6}-C_{7})\\ V_{z1}=1-V_{z0}=1-(\frac{1}{2}A-\frac{1}{2}S_{2}+C_{4}-E_{3}-R+Ca+Cb-Em-En-C_{5}+E4)/(E_{6}-C_{7}) \end{array}$$

Inference 3: As financial support from local governments to cooperatives, the benefits of ecological product production, the costs of traditional product production, the premiums from product promotion, economies of scale in production, social benefits, and the revenue from ecological tourism projects increase, cooperatives will not only have sufficient funds, the benefits of ecological product production and related extension projects will increase, and cooperatives will have more benefits to pay dividends to members. At this time, cooperatives choose ecological product production strategies; Conversely, when the costs of ecological product production decrease, along with the benefits of traditional product production, expenses for ecological product certification, and costs related to product promotion, technical training, and ecological tourism projects, it shows that the cost of ecological product production by cooperatives will be reduced. And in the case of low cost, its production strategy is consistent with the policy requirements, which will have a positive effect on the ecological environment. At this time, the cooperative chooses the production strategy of ecological products.

Proof: By taking the first-order partial derivatives of the probability Vz1Vz1Vz1 of the cooperative adopting the ecological product production strategy with respect to S_2_、C_4_、E_3_、A、C_5_、E_4_、R、E_m_、E_n_、C_a_、C_b_、C_7_ and E_6,_


$$\begin{array}{c} \partial V_{z1}/\partial S_{2}>0,\partial V_{z1}/\partial E_{3}>0,\;\partial V_{z1}/\partial C_{5}>0,\;\partial V_{z1}/\partial R>0,\partial V_{z1}/\partial Em>0,\partial V_{z1}/\partial En>0\;\\ \partial V_{z1}/\partial E_{6}>0,\partial V_{z1}/\partial C_{4}<0,\;\partial V_{z1}/\partial E_{4}<0,\partial V_{z1}/\partial A>0,\partial V_{z1}/\partial Ca>0,\partial V_{z1}/\partial Cb>0,\partial V_{z1}/\partial C_{7}>0. \end{array}$$

#### 3.2.4. Analysis of member strategy stability.

The expected benefits for members participating in “ecological product production” and “traditional product production” are provided by E41 and E42, respectively:


$$\begin{array}{c} E_{41}=xyzd_{1}+xz(1-y)d_{5}+yz(1-x)d_{9}+z(1-x)(1-y)d_{13}+xy(1-z)d_{3}+x(1-y)(1-z)d_{7}+\\ y(1-x)(1-z)d_{11}+(1-x)(1-y)(1-z)d_{15}\\ E_{42}=xyzd_{2}+xz(1-y)d_{6}+yz(1-x)d_{10}+z(1-x)(1-y)d_{14}+xy(1-z)d_{4}+x(1-y)(1-z)d_{8}+\\ y(1-x)(1-z)d_{12}+(1-x)(1-y)(1-z)d_{16} \end{array}$$

The average expected benefit is: ${\overset{―}{E}}_{4}=mE_{41}+(1-m)E_{42}$

The dynamic equation is expressed as:


$$F(m)=m(E_{41}-{\overset{―}{E}}_{4})=m(1-m)(E_{41}-E_{42})=m(1-m)\tau \left(x,y,z\right)$$

$$\tau (x,y,z)=yzF+zU_{1}+zB+rU_{2}-2zrU_{2}-U_{2}+zU_{2}$$

From the above equations, it is evident that the factors influencing member strategies primarily include the probability of decisions made by other entities, the additional benefits members receive from ecological tourism projects, the benefits obtained from participating in ecological and traditional product production, the benefits received when members’ production intentions do not align with the cooperative, and the skill improvements gained by members. For members’ participation in the ecological product production strategy to remain stable, the following conditions must be satisfied: F(z)=0且F’(z)<0.

Let $z_{0}=(U_{2}-rU_{2})/(yF+U_{1}+B-2rU_{2}+U_{2})$, When z = z0, F(m)=0, making all m∈[0,1] stable points. When zz0, from F(m)=0, it can be deduced that members participating in “ecological product production” (m = 1) and “traditional product production” (m = 0) are both stable strategies.

**Fig 4:**
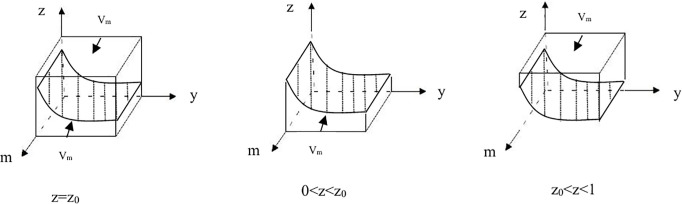
Phase diagram of membership strategy. **(A)**The phase diagram of membership strategywhen z = z_0._ **(B)**The phase diagram of membership strategywhen 0 < z < z_0._ **(C)**The phase diagram of membership strategywhen z_0_ < z < 1.

Proposition 4: For members’ stable strategy to be “traditional product production,” the condition 0 < z < z_0_ must be satisfied. For the stable strategy to be “ecological product production,” the condition z_0_ < z < 1 needs to be fulfilled. If z = z_0_, the stability of the strategy cannot be determined.Based on the above analysis, the phase diagram of the cooperative strategy can be obtained.See Fig 4A–C.

V_m0_ and V_m1_ represent the probabilities of members participating in “traditional product production” and “ecological product production,” respectively.


$$\begin{array}{c} V_{m0}=\int_{0}^{1}\int_{0}^{1}((U_{2}-rU_{2})/(yF+U_{1}+B-2rU_{2}+U_{2}))dydm=[(U_{2}-rU_{2})/F]log(yF+U_{1}+B-2rU_{2}+U_{2})\\ V_{m1}=1-Vm0=1-[(U_{2}-rU_{2})/F]log(yF+U_{1}+B-2rU_{2}+U_{2}) \end{array}$$

Inference 4: When the benefits members receive from participating in ecological product production, the additional benefits from ecological tourism projects, and skill improvements increase, not only will members receive more dividends from cooperatives, but they can also improve their production skills by participating in the production of ecological products, members will choose to participate in ecological product production. Conversely, when the benefits from participating in traditional product production decrease, members opt for ecological product production.

Proof: $\partial V_{m1}/\partial U_{1}>0,\partial V_{m1}/\partial F>0,\partial V_{m1}/\partial B>0,\partial V_{m1}/\partial U_{2}<0$

### 3.3 Strategy combination stability analysis

The following illustrates the formation conditions and process of stable strategies in the four-party game among the government, enterprises, cooperatives, and members by constructing the replicator dynamic equations.


$$\{\begin{array}{c} {}*20cF(x)=x(1-x)\phi (y,z,m)\\ F(y)=y(1-y)\theta (x,z,m)\\ F(z)=z(1-z)\rho (x,y,m)\\ F(m)=m(1-m)\tau (x,y,z) \end{array}$$

By simultaneously solving the replicator dynamic equations for the four parties, sixteen feasible solutions are derived. To analyze the stability of these sixteen pure strategy equilibrium solutions, Lyapunov’s first method is employed. The application of this method provides a deeper understanding of the stability characteristics of these equilibrium solutions within a dynamic system. The stability analysis focuses on the eigenvalues of the Jacobian matrix. If all the real parts of the eigenvalues are negative, the equilibrium point is defined as an asymptotically stable point. If at least one eigenvalue has a real part that is positive, the equilibrium point is unstable. Additionally, if the Jacobian matrix has eigenvalues with zero real parts and the rest have negative real parts, the equilibrium point is in a critical state, and its stability cannot be determined solely by the eigenvalue signs. To gain a comprehensive understanding of the system’s stability and its evolutionary strategies, a local stability analysis of the Jacobian matrix is conducted to identify evolutionary stable strategies. Furthermore, from various cooperative strategy perspectives, the evolutionary paths and stability of strategy combinations among game participants can be explored in greater depth. The Jacobian matrix mentioned is shown below:


$$J=\left[\begin{array}{cccc} {}*20c\partial F(x)/\partial x & \partial F(x)/\partial y & \partial F(x)/\partial z & \partial F(x)/\partial m\\ \partial F(y)/\partial x & \partial F(y)/\partial y & \partial F(y)/\partial z & \partial F(y)/\partial m\\ \partial F(z)/\partial x & \partial F(z)/\partial y & \partial F(z)/\partial z & \partial F(z)/\partial m\\ \partial F(m)/\partial x & \partial F(m)/\partial y & \partial F(m)/\partial z & \partial F(m)/\partial m \end{array}\right]$$

$$J=\left[\begin{array}{cccc} @c@c@c@c@\begin{array}{c} (1-2x)(-C_{1}-zS_{2}+zA\\ -yS_{1}+yA+E_{a}+E_{c}) \end{array} & x(1-x)(A-S_{1}) & x(1-x)(A-S_{2}) & 0\\ {}[6pt]y(1-y)(A-S_{1}) & \begin{array}{c} (1-2y)(zE_{5}-zC_{6}+xS_{1}-xA\\ -C_{2}+C_{3}+E_{1}+E_{p}-E_{2}) \end{array} & y(1-y)(E_{5}-C_{6}) & 0\\ {}[6pt]z(1-z)(S_{2}-A) & z(1-z)(E_{6}-C_{7}) & \begin{array}{c} (1-2z)(yE_{6}-yC_{7}+xS_{2}\\ -xA-C_{4}+E_{3}+R-C_{a}\\ -C_{b}+E_{m}+E_{n}+C_{5}-E_{4}) \end{array} & 0\\ {}[6pt]0 & m(1-m)zF & \begin{array}{c} m(1-m)(yF+U_{1}\\ +B-2rU_{2}+U_{2}) \end{array} & \begin{array}{c} (1-2m)(yzF+zU_{1}\\ +zB+rU_{2}-2zrU_{2}\\ -U_{2}+zU_{2}) \end{array} \end{array}\right]$$

#### 3.3.1 Analysis of asymptotic stability of equilibrium points under the cooperative’s traditional product production strategy.

When the cooperative maintains the “traditional product production” state, the condition for ensuring its continuity is E_31_-E_32_ < 0. The stability of each equilibrium point under this scenario is detailed in Table 2.

**Table 2:** Asymptotic stability of equilibrium points under the cooperative’s traditional product production strategy.

Equilibrium Point	Eigenvalue				Symbol	Stability	Condition
	λ1				λ2	λ3	λ4
(0,0,0,0)	rU_2_-U_2_	E_a_-C_1_ + E_c_	C_3_-C_2_ + E_1_-E_2_ + E_p_	C_5_-C_4_-C_a_-C_b_ + E_3_- E_4_ + E_m_ + E_n_ + R	-, + ,-,-	×	
(1,0,0,0)	rU_2_-U_2_	C_1_-E_a_-E_c_	C_3_-C_2_-A + E_1_-E_2_ + E_p_ + S_1_	C_5_-C_4_-A-C_a_-C_b_ + E_3_- E_4_ + E_m_ + E_n_ + R + S_2_	-,-,-,-	ESS	①
(0,1,0,0)	rU_2_-U_2_	A-C_1_ + E_a_ + E_c_- S_1_	C_2_-C_3_-E_1_ + E_2_-E_p_	C_5_ -C_4_-C_7_-C_a_-C_b_ + E_3_-E_4_ + E_6_ + E_m_ + E_n_ + R	-, + , + ,*	×	
(0,0,0,1)	U_2_-rU_2_	E_a_-C_1_ + E_c_	C_3_-C_2_ + E_1_-E_2_ + E_p_	C_5_-C_4_-C_a_-C_b_ + E_3_- E_4_ + E_m_ + E_n_ + R	+, + ,-,-	×	
(1,1,0,0)	rU_2_-U_2_	C_1_-A-E_a_-E_c_ + S_1_	A + C_2_-C_3_-E_1_ + E_2_-E_p_- S_1_	C_5_-C_4_-A-C_7_-C_a_-C_b_ + E_3_-E_4_ + E_6_ + E_m_ + E_n_ + R + S_1_	-,-, + ,*	×	
(1,0,0,1)	U_2_-rU_2_	C_1_-E_a_-E_c_	C_3_-C_2_-A + E_1_-E_2_ + E_p_ + S_1_	C_5_-C_4_-A-C_a_-C_b_ + E_3_- E_4_ + E_m_ + E_n_ + R + S_1_	+,-,-,-	×	
(0,1,0,1)	U_2_-rU_2_	A-C_1_ + E_a_ + E_c_- S_1_	C_2_-C_3_-E_1_ + E_2_-E_p_	C_5_ -C_4_-C_7_-C_a_-C_b_ + E_3_-E_4_ + E_6_ + E_m_ + E_n_ + R	+, + , + ,*	×	
(1,1,0,1)	U_2_-rU_2_	C_1_-A-E_a_-E_c_ + S_1_	A + C_2_-C_3_-E_1_ + E_2_-E_p_-S_1_	C_5_-C_4_-A-C_7_-C_a_-C_b_ + E_3_-E_4_ + E_6_ + E_m_ + E_n_ + R + S_2_	+,-, + ,*	×	

Note: “+” means that the eigenvalue is positive, “-” means that the eigenvalue is negative, “*” means that the eigenvalue is uncertain, and “×” means that the equilibrium point is unstable.

Condition: ①C_1_-E_a_-E_c_ < 0, C_3_-C_2_-A + E_1_-E_2_ + E_p_ + S_1_ < 0, C_5_-C_4_-A-C_a_-C_b_ + E_3_-E_4_ + E_m_ + E_n_ + R + S_2_ < 0.

When the cooperative produces traditional products, there is a strategy combination (1,0,0,0), which is a stable point. When C_1_-Ea-Ec < 0、C_3_-C_2_-A + E_1_-E_2_ + Ep + S_1_ < 0、C_5_-C_4_-A-Ca-Cb + E_3_-E_4_ + Em + En + R + S_2_ < 0, the social reputation benefits the government gains from implementing incentive policies and the social reputation losses from inaction are greater than the costs of implementing certification policies. Thus, the government will choose to implement incentive policies. However, for enterprises and cooperatives, the costs of ecological product certification are high. The costs of traditional product production are lower than those of ecological product production, and the revenue from traditional product production is higher than that from ecological product production. Additionally, when cooperatives engage in ecological product production, they incur higher costs for brand promotion and hiring experts. As a result, enterprises will choose to engage in traditional product operations, and cooperatives will opt for traditional product production. When cooperatives abandon ecological product production, members will also choose traditional product production, as they cannot benefit from the convenience provided by the cooperative’s integration of ecological resources.

#### 3.3.2 Analysis of asymptotic stability of equilibrium points under the cooperative’s ecological product production strategy.

When the cooperative maintains the “ecological product production” state, the condition for ensuring its continuity is E_31_-E_32_ > 0. The stability of each equilibrium point under this scenario is detailed in Table 3.

**Table 3:** Asymptotic stability of equilibrium points under the cooperative’s ecological product production strategy.

Equilibrium Point	Eigenvalue				Symbol	Stability	Condition
	λ1				λ2	λ3	λ4
(0,0,1,0)	B + U_1_-rU_2_	A-C_1_ + E_a_ + E_c_-S_1_	C_3_-C_2_-C_6_ + E_1_-E_2_ + E_5_ + E_p_	C_4_ -C_5_ + C_a_ + C_b_-E_3_ + E_4_ -E_m_-E_n_-R	+,-,-,-	×	
(1,0,1,0)	B + U_1_-rU_2_	C_1_-A-E_a_-E_c_ + S_2_	C_3_-C_2_-A-C_6_ + E_1_-E_2_ + E_5_ + E_p_ + S_1_	A + C_4_-C_5_ + C_a_ + C_b_-E_3_ + E_4_-E_m_-E_n_-R-S_2_	+, + ,-,-	×	
(0,1,1,0)	B + F + U_1_-rU_2_	2A-C_1_ + E_a_ + E_c_-S_1_-S_2_	C_2_-C_3_ + C_6_-E_1_ + E_2_- E_5_-E_p_	C_4_-C_5_ + C_7_ + C_a_ + C_b_-E_3_ + E_4_-E_6_-E_m_-E_n_-R	+,-,-,-	×	
(0,0,1,1)	rU_2_-U_1_-B	A-C_1_ + E_a_ + E_c_-S_2_	C_3_-C_2_-C_6_ + E_1_-E_2_ + E_5_ + E_p_	C_4_ -C_5_ + C_a_ + C_b_-E_3_ + E_4_ -E_m_-E_n_-R	-,-,-,-	ESS	①
(1,1,1,0)	B + F + U_1_-rU_2_	C_1_-2A-E_a_-E_c_ + S_1 _+ S_2_	A + C_2_-C_3_ + C_6_-E_1_ + E_2_-E_5_-E_p_-S_1_	A + C_4_-C_5_ + C_a_ + C_b_-E_3_ + E_4_-E_m_-E_n_-R-S_2_	+, + , + ,-	×	
(1,0,1,1)	rU_2_-U_1_-B	C_1_-A-E_a_-E_c_ + S2	C_3_-C_2_-A-C_6_ + E_1_-E_2_ + E_5_ + E_p_ +S_1_	A + C_4_-C_5_ + C_a_ + C_b_-E_3_ + E_4_-E_m_-E_n_-R-S_2_	-,-,-,-	ESS	②
(0,1,1,1)	rU_2_-U_1_-B-F	2A-C_1_ + E_a_ + E_c_-S_1_-S_2_	C_2_-C_3_ + C_6_-E_1_ + E_2_- E_5_-E_p_	C_4_-C_5_ + C_7_ + C_a_ + C_b_-E_3_ + E_4_-E_6_-E_m_-E_n_-R	-,-, + ,-	×	
(1,1,1,1)	rU_2_-U_1_-B-F	C_1_-2A-E_a_-E_c_ + S_1 _+ S_2_	A + C_2_-C_3_ + C_6_-E_1_ + E_2_-E_5_-E_p_-S_1_	A + C_4_-C_5_ + C_7_ + C_a_ + C_b_-E_3_ + E_4_-E_m_-E_n_-E_6_-R-S_2_	-, + , + ,*	×	

Note: “+” means that the eigenvalue is positive, “-” means that the eigenvalue is negative, “*” means that the eigenvalue symbol is uncertain, and “×” means that the equilibrium point is unstable.

Conditions: ①A-C_1_ + E_a_ + E_c_-S_2_ < 0, C_3_-C_2_-C_6_ + E_1_-E_2_ + E_5_ + E_p_ < 0, C_4_-C_5_ + C_a_ + C_b_-E_3_ + E_4_-E_m_-E_n_-R < 0; ②C_1_-A-E_a_-E_c_ + S_2_ < 0, C_3_-C_2_-A-C_6_ + E_1_-E_2_ + E_5_ + E_p_ + S_1_ < 0, A + C_4_-C_5_ + C_a_ + C_b_-E_3_ + E_4_-E_m_-E_n_-R-S_2_ < 0.

When the cooperative produces ecological products, there is a strategy combination (0,0,1,1) and (1,0,1,1), which is a stable point. When A-C_1_ + E_a_ + E_c_-S_2_ < 0、C_3_-C_2_-C_6_ + E_1_-E_2_ + E_5_ + E_p_ < 0、C_4_-C_5_ + C_a_ + C_b_-E_3_ + E_4_-E_m_-E_n_-R < 0. The higher costs of governments when conducting incentive policies, the financial support given to cooperatives is also higher and the certification cost is lower, this makes the government choose the inaction strategy; for businesses, its ecological product operation has higher cost and lower income, for companies focused on profit, they will choose to operate their traditional products; for the cooperatives, its production of ecological products has lower costs and higher benefits, and the production of ecological products will obtain higher economies of scale benefits and social benefits, therefore, cooperatives choose to produce ecological products; when the cooperatives conducts ecological product production, experts will be hired to train members and integrate ecological resources, this is more beneficial to the commune members, therefore, the commune members will also choose to participate in the production of ecological products, that is, (0, 0,1, 1) is the stability point. When C_1_-A-E_a_-E_c_ + S_2_ < 0、C_3_-C_2_-A-C_6_ + E_1_-E_2_ + E_5_ + E_p_ + S_1_ < 0、A + C_4_-C_5_ + C_a_ + C_b_-E_3_ + E_4_-E_m_- E_n_ -R-S_2_ < 0, the certification cost of the incentive policy is lower, the cost of ecological product certification is higher, and the social reputation benefits of the incentive strategy is higher.The social reputation loss is high, so the government chooses to implement incentive policies; for enterprises, they still choose to operate traditional products on the premise of obtaining the maximum profit; cooperatives produce ecological products and have higher scale and social benefits of the government, so the cooperative society chooses to produce ecological products on the basis of the cooperative strategy, members choose to participate in the production, thus (1,0,1,1) is stable.

## 4. Simulation analysis

### 4.1 Parameter assignment

To intuitively demonstrate the evolutionary process of the behavioral strategies of the game participants and to verify the validity of the constructed model, numerical simulations of different model parameters were conducted using Matlab. The stable strategies of the four parties in the evolutionary game were analyzed. According to the relevant research and practical cases of the market mechanism of ecological products, the basis of parameter assignment can be summarized as follows:

With reference to the relevant documents of ecological product certification, county-level governments need to invest in basic costs such as policy formulation and personnel training to carry out certification. The average annual cost of project management in small counties is 1–2 million yuan, and C_1_ is set to 1. The pomelo in Changshan County, Zhejiang Province is a national geographical indication product in China. The cost of ecological product production by enterprises will be 50% −80% higher than that of the traditional model. Taking Zhejiang Aijia Fruit and Vegetable Development Co., Ltd.as an example, the company sells Changshan pomelo and processes Changshan pomelo into fruit juice. The cost of purchasing ecologically grown pomelo is about CNY 3/ jin. After processing, the cost is about CNY 8.7/ jin, while the traditionally grown pomelo is about CNY 5.8/ jin.The premium rate of enterprise ecological products is about 30% −40%. After processing into fruit juice, the sales price of ecologically planted grapefruit is 11.94 yuan/ bottle. Here, E1 = 11, corresponding to E_2_ = 8. Changshan Cooperative is the main base for the planting of pomelo. The planting cost of ecological pomelo is 60% higher than that of traditional planting of 2000 yuan/ mu, about 3200 yuan/ mu. After standardization, it is 2 and 3.2. After removing the infrared of farmers, the collective income of the village is about 900,000 yuan, and the income of ecological products is 50% higher than that of traditional planting. Therefore, the traditional planting is about 60,000 yuan, and 9 and 6 are taken after standardization. For ecotourism projects, ‘ Liangshan Cooperative ‘ cooperates with Tengyun Company to build a modern tourism base in Jinyuan Village, Dongan Township, in accordance with the model of ‘ one village is a hotel ‘. The ‘ Liangshan Cooperative ‘ has invested in the stock of idle residential housing management rights, Tengyun Company has invested in homestay renovation and transformation, and government supporting funds have been used to transform and upgrade infrastructure. The cost of the investment of enterprises and cooperatives is not clearly indicated. It is converted to 550,000 yuan and 600,000 yuan by referring to the relevant shareholding methods. At the end of 2021, its economic income is about 5 million yuan. According to the proportion of shares, E_5_ is 1.2 and E_6_ is 1.6. Brand promotion costs usually account for 3% −5% of sales, which is set to 0.3, reflecting the moderate investment of cooperatives in brand building. The per capita cost of inviting experts to train members in agricultural cooperatives is about 800 yuan/ year, which is 0.8 after standardization. The government ‘s promotion of ecological certification can improve public satisfaction, and the value is often assigned to 2 in quantitative research. According to the Ecological Environment Research Center of the Chinese Academy of Sciences, the environmental benefits of ecological agriculture to reduce pesticide use are estimated to be about 1.5 yuan/ mu. With reference to the public policy evaluation model, the loss of social trust caused by government inaction is about 40% of the positive return, so set 0.8. The government ‘s subsidy for technological transformation of ecological enterprises shall not exceed the general standard of 30% of investment and 35% of subsidy for cooperative facilities construction. Therefore, S_1_ and S_2_ are set at 30 and 35. According to the charge standard of China Green Food Development Center, the certification fee of production base under 500 acres is about 6400 yuan, which is converted into model unit 0.64. The remaining parameters are set by referring to the relevant literature.The specific parameter values are shown in **Table 4**.

**Table 4:** Parameter assignment.

Parameter	C1	C_2_	C_3_	C_4_	C_5_	C_6_	C_7_	C _a_	C_b_	S_1_	S_2_	A	E_1_	E_2_	E_3_
Assigned Value	1	8.7	5.8	3.2	2	55	60	0.3	0.8	30	35	0.64	11	8	9
Parameter	E_4_	E_5_	E_6_	E_m_	E_n_	E_p_	E_a_	E_b_	E_c_	F	R	B	r	U_1_	U_2_
Assigned Value	6	1.2	1.6	2.2	1.3	1	2	1.5	0.8	0.9	2.3	0.8	0.5	5.4	3.6

### 4.2 The impact of initial strategies on system evolution

Under the premise that other parameters remain constant, the values of x, y, z, and m are set to one of the values from the set {0.3,0.5,0.7,0.9}. Based on these settings, the evolutionary process of the strategies for the four participants was analyzed, and the results are presented in graphical form.

**Fig 5:**
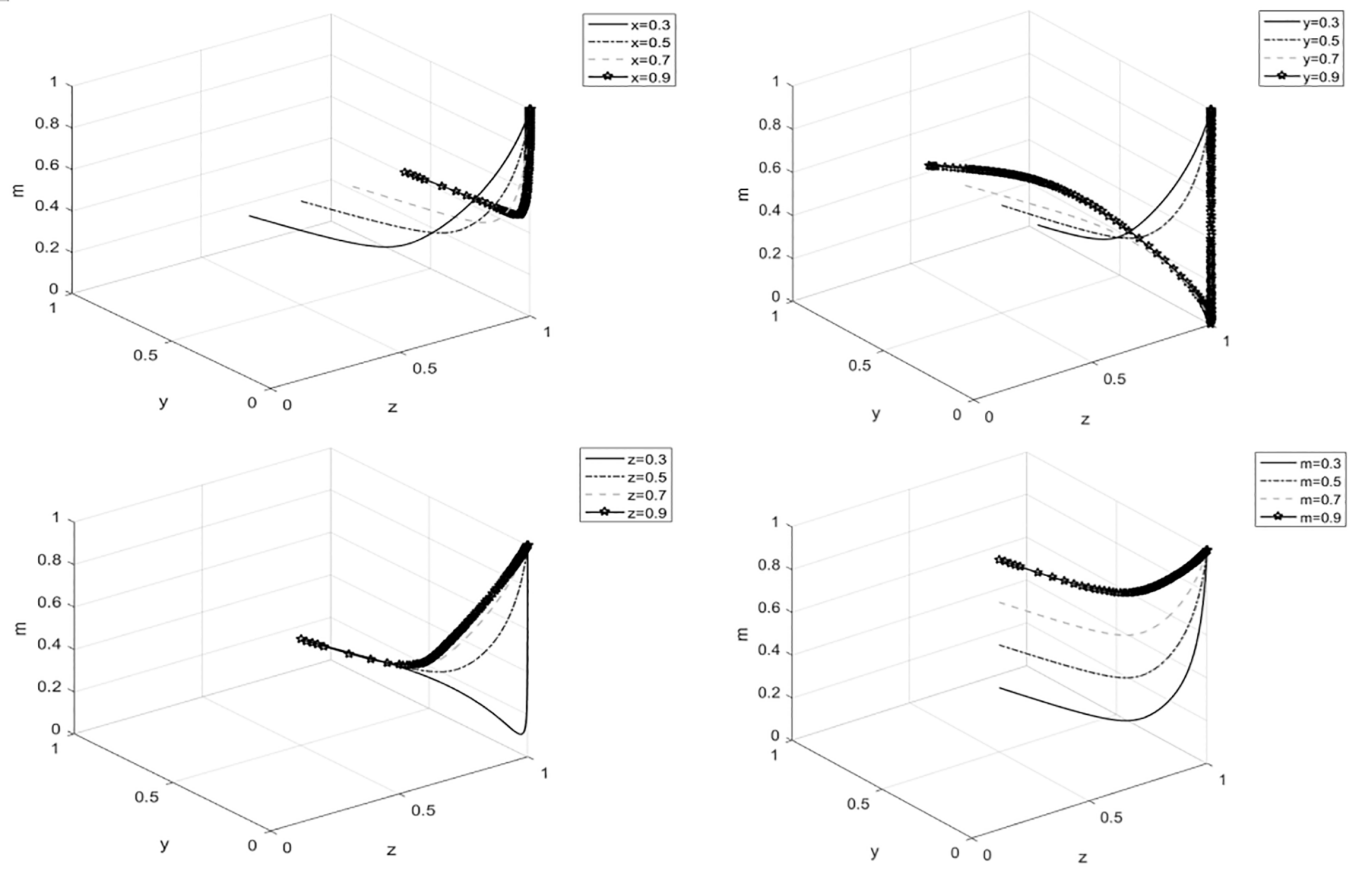
The impact of initial strategies on system evolution. **(A)** The impact of the local government’s initial strategy choice on system evolution. **(B)**The impact of the enterprise’s initial strategy choice on system evolution. **(C)**The impact of the cooperative’s initial strategy choice on system evolution. **(D)**The impact of the cooperative members’ strategy choice on system evolution.

From Fig 5, it can be observed that in the game system, as the initial strategies of the participants increase, the system exhibits complex dynamics. For enterprises,see Fig 5B, as the initial strategy probability of cooperatives gradually increases, the probability of enterprises choosing the “traditional product management” strategy shows a decreasing trend, and government influence on this is not significant. The reason is that in reality, ecological products are a new concept of green products, and the market acceptance during the initial promotion phase is not yet clear. Therefore, under the premise of profitability, enterprises tend to favor traditional agricultural products with a clearer market. However, as the cooperative’s initial strategy increases, their willingness to produce ecological products strengthens, allowing enterprises to see the market potential of ecological products, thus reducing the probability of choosing the “traditional product management” strategy. For cooperatives,see Fig 5C, the probability of choosing the “ecological product production” strategy increases as the initial strategy probabilities of the other three participants increase. For cooperative members,see Fig 5D, as the cooperative’s initial strategy increases, the probability of choosing to “participate in ecological product production” increases. However, as the enterprise’s initial strategy increases, the speed of this evolution decreases. Government influence on this is not significant. For the local government,see Fig 5A, when the other three participants all choose strategies related to traditional products, the local government tends to adopt the “incentive” strategy to encourage the other participants to engage in the production and management of ecological products. As their willingness to participate in ecological product production increases, the government’s strategy will shift towards “inaction.”

### 4.3 The impact of parameter changes on system evolution

#### 4.3.1 The impact of ecological product certification fees a on system evolution.

**Fig 6:**
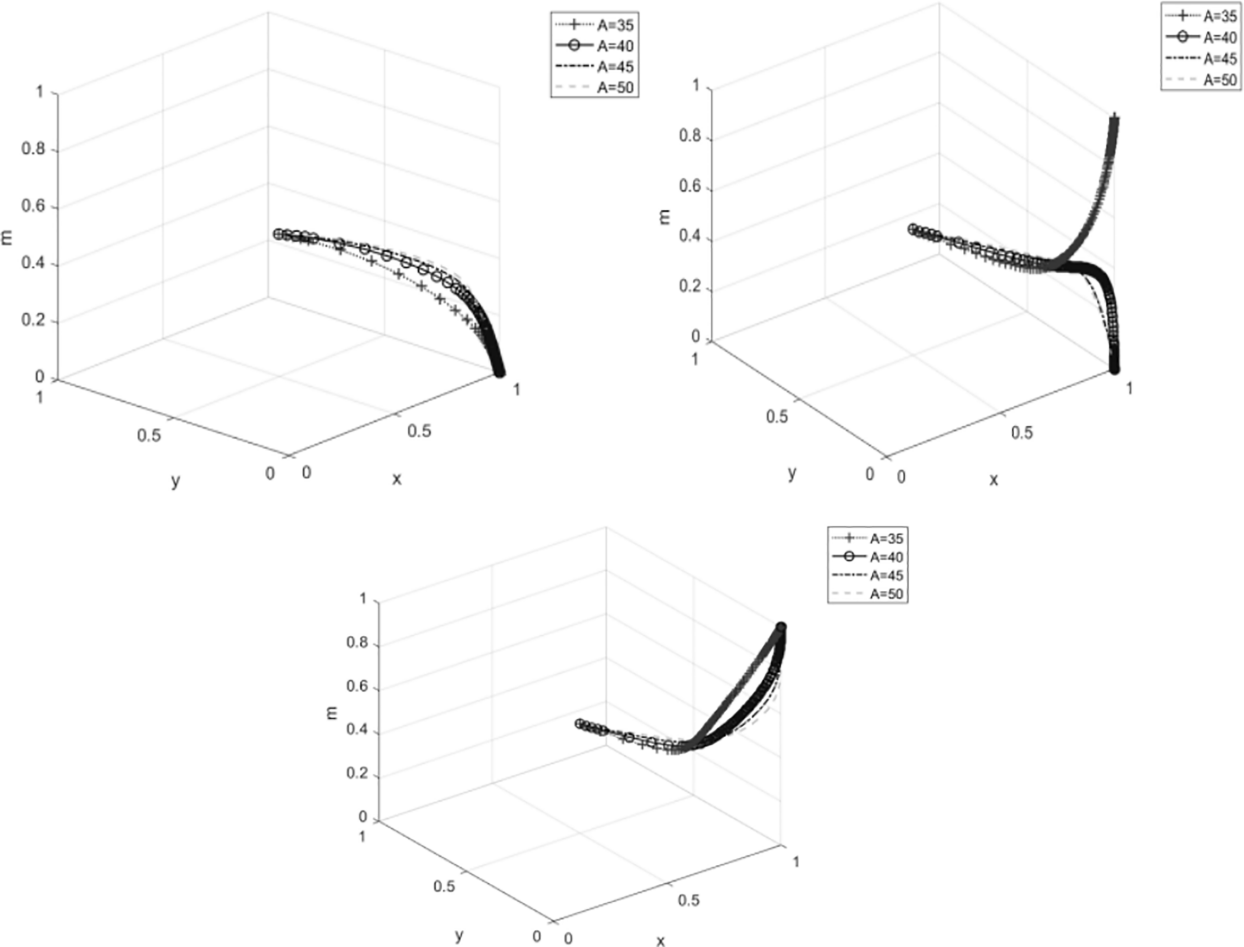
The impact of ecological product certification fees on system evolution. **(A)**The impact of ecological product certification fees on system evolution when z = 0. **(B)**The impact of ecological product certification fees on system evolution when z = 0.5. **(C)**The impact of ecological product certification fees on system evolution when z = 1.

The changes in ecological product certification fees have a complex impact on the strategies of the game participants. When cooperatives choose not to produce ecological products,see Fig 6A, as certification fees increase, the speed at which the government evolves towards the “incentive” strategy increases, and enterprises, due to the rising costs, will opt for “traditional product management.” When cooperatives’ willingness to participate increases,see Fig 6B, members will choose to “participate in ecological product production” when certification fees are lower. When cooperatives choose to produce ecological products,see Fig 6C, the members’ strategies also evolve towards “participation in ecological product production.” As certification fees increase, the speed at which the government evolves towards the “incentive” strategy increases, but the speed at which members evolve towards “participation in ecological product production” slows down. At this time, the evolution speed of enterprises is not significant, possibly because cooperative participation reduces the impact of certification fees on enterprises.

#### 4.3.2 The impact of government financial support s_2_ to cooperatives on system evolution.

**Fig 7:**
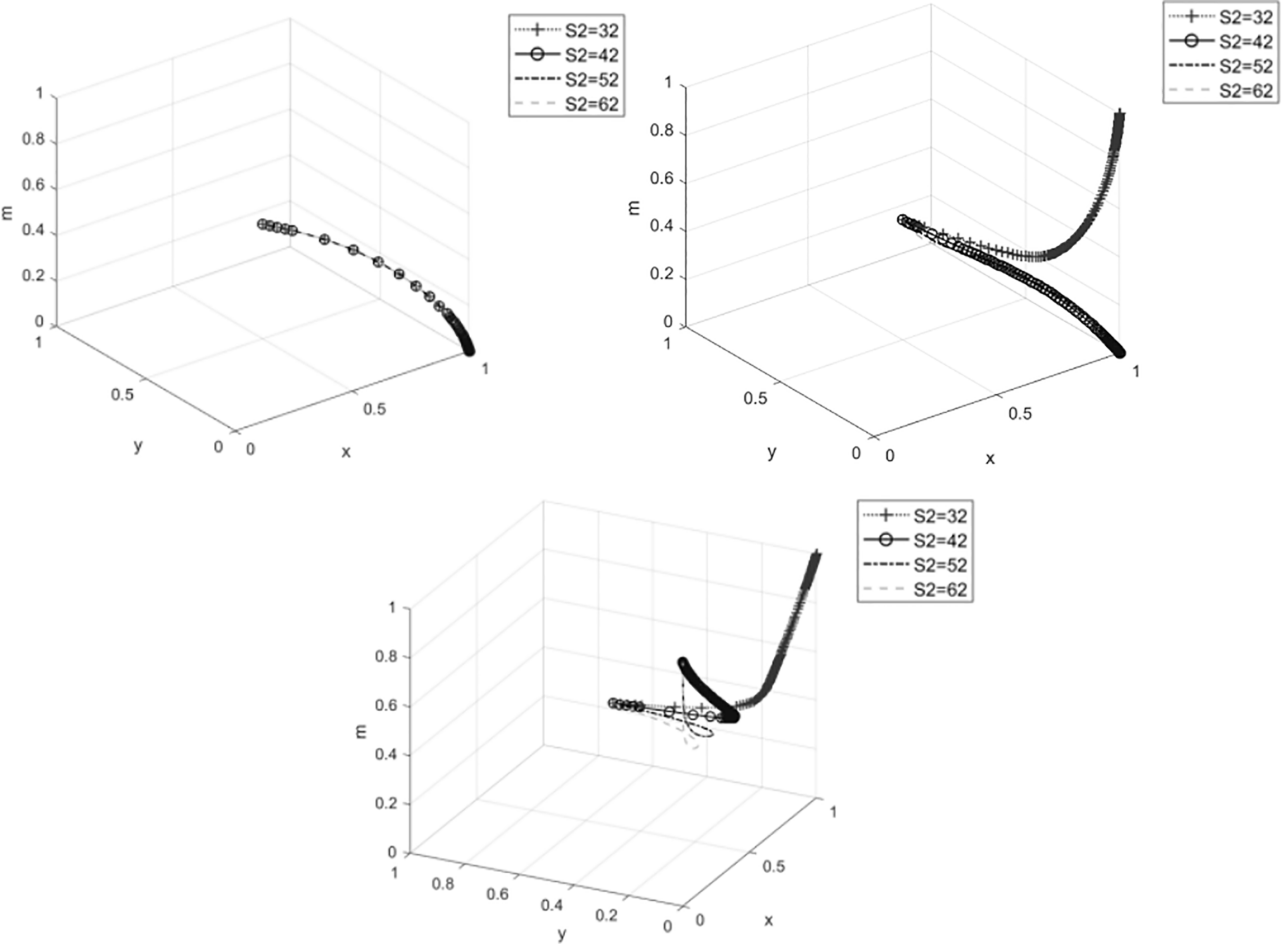
The impact of government financial support to cooperatives on system evolution. **(A)**The impact of government financial support to cooperatives on system evolution when x = 0. **(B)**The impact of government financial support to cooperatives on system evolution.

Fig 7A–C shows that when cooperatives choose to produce traditional products, the financial support provided by the government to cooperatives does not significantly affect the strategies of the participants. However, as the willingness of cooperatives to participate increases, the increase in financial support accelerates the evolution of enterprises towards the “traditional product management” strategy. The reason is that when the government provides high financial support to cooperatives, the technical support for enterprises may be reduced, thus weakening their willingness to manage ecological products. When the cooperative’s probability of engaging in ecological product production reaches 1, the government will choose the “incentive” strategy when financial support is low, but as financial support increases, the government’s strategy will evolve towards “inaction.” This is because when cooperatives have a strong willingness to produce ecological products, the government does not need to implement the “incentive” strategy to encourage participants to engage in ecological product production. For cooperative members, the speed at which they evolve towards the “participation in ecological product production” strategy also accelerates.

when x = 0.5. (C)The impact of government financial support to cooperatives on system evolution

when x = 1

## 5. Case study

Most of the ecological resources are in a decentralized state, and the management and use rights of these ecological resources are mostly in the hands of farmers. The decentralized operation of these ecological resources hinders the process of payment for ecosystem services. The ‘ Liangshan Cooperative ‘ in Zhejiang Province is an ecological product management platform that adopts a ‘ decentralized input and centralized output ‘ model. Taking the Liangshan Cooperative in Changshan County as an example, before the establishment of the cooperative, the local fruit growers of Huyou were all independent, and the quality of Huyou in the planting area was uneven, resulting in Huyou often being unsalable. After the establishment of the cooperative, it sought enterprises, linked farmers, and met the market, trying to standardize the orchard planting reform, change its decentralized management status, and form a sales alliance with intentional orchards, which not only improved the quality of the orchard, but also solved its unsalable problem. At present, Changshan Huyou has become China ‘s national geographical indication product.In addition to orchard planting, the two-mountain cooperative also played a very important role in terrace restoration. The two-mountain cooperative in Yunhe County introduced the planting of foreign contractors through investment promotion, and implemented unified planning and unified standards, so that the county systematically repaired more than 5,000 mu of terraces, and the abandonment rate decreased from 45% in 2016 to 3.3% at present. In Zhejiang Province, there are many such examples. The establishment of the ‘ two mountain cooperatives ‘ has made the ecological resources no longer in a decentralized state, and its multi-sectoral linkage with the government and related enterprises has made the operation and production of ecological products. Higher income has also promoted the realization of payment for ecosystem services.

## 6. Conclusion and policy recommendations

### 6.1 Conclusion

By constructing a four-party evolutionary game model involving local government, enterprises, cooperatives, and cooperative members, this study deeply analyzed the strategy stability of these participants and the stability of strategy combinations. Parameter assignments and simulation analyses were conducted to better understand the dynamic changes and stable states of these strategies. The key conclusions are as follows:

First, the main factors influencing the local government’s strategy are ecological product certification fees, the cost of conducting ecological product certification, the technical support provided by the government to enterprises, financial support provided to cooperatives, social reputation benefits, and social reputation losses. The key factors influencing enterprise strategies are the technical support provided by the government, the costs of managing various types of products, and the sales revenue generated from those products. Cooperative strategies are influenced by certification fees, government financial support, the costs and benefits of producing various products, the costs and benefits of eco-tourism projects, economies of scale, and social benefits. The factors affecting cooperative members’ strategies mainly include the additional benefits gained from eco-tourism projects and the profits from participating in the production of both ecological and traditional products.

Second, in strategy analysis, it is observed that the stable equilibrium points differ under different cooperative strategies. When the cooperative adopts the “ traditional products” strategy, the stable equilibrium point for the strategy combination is (1,0,0,0). However, when the cooperative shifts to the “ ecological products.” strategy, the equilibrium points change to (0,0,1,1) and (1,0,1,1), indicating how different cooperative strategies impact the stability of strategy combinations among the game participants.

Third, the analysis reveals that among the four participants, the enterprise’s strategy is most significantly influenced by changes in the cooperative’s initial strategy probabilities. The stability of the cooperative’s strategy is affected by the other three participants, and the cooperative members’ strategy choices are also primarily influenced by changes in the cooperative’s initial strategy probabilities.

Finally, the factors affecting system equilibrium include ecological product certification fees and the financial support provided by the government to cooperatives. The system will evolve towards the stable strategies of “Incentive–Traditional Product Management–Ecological Product Production–Participation in Ecological Product Production” or “Inaction–Traditional Product Management–Ecological Product Production–Participation in Ecological Product Production.” Regardless of the evolutionary outcome, the cooperative’s strategy ultimately remains focused on ecological product production, indicating that cooperatives play a key role in the realization of the value of ecological products.

### 6.2 Policy recommendations

Based on the above conclusions, the following policy recommendations are proposed:

First, the local government should increase technical support and financial assistance to enterprises and cooperatives, while also improving relevant policies and regulations(Wang Kuifeng et al., 2023) [23]. Measures such as tax incentives and financial subsidies should be provided to stimulate the enthusiasm of cooperatives and farmers. Additionally, environmental education should be strengthened to raise awareness of ecological protection among cooperatives, members, and consumers, creating a societal environment where ecological products are widely supported and valued.

Second, enterprises, while pursuing profits, should adopt a green development mindset. They should actively respond to the government’s call by incorporating the production and management of ecological products into their development strategies. Enterprises should strengthen their collaboration with cooperatives and members to jointly promote the production and management of ecological products, rather than merely focusing on high profits.

Third, cooperatives, with government support, should encourage members to participate in the production of ecological products by providing training and technical assistance to improve their production skills. Cooperatives should also enhance internal management and training to raise overall capacity and quality. Furthermore, they should actively engage in market promotion, expanding sales channels, and increasing the visibility and market share of their products. Branding efforts should also be emphasized to build locally distinctive ecological product brands and to improve the reputation and recognition of their products.

Finally, cooperative members should embrace green concepts and actively participate in the production of ecological products. They should focus on learning scientific planting and breeding techniques, enhancing their technical skills, and understanding the multiple values of ecological products. Members should also provide valuable suggestions and feedback to the cooperative to enhance production and management strategies.

**Attention**

1. All research in this paper is carried out within the relevant ethical and legal framework,
2. The data of this paper will be uploaded to the supporting information along with the manuscript,
3. This research was supported by the National Social Science Foundation of China under the project “Research on the Mechanism of Farmers’ Cooperatives Promoting the Realization of Ecological Product Value in Southwest Ethnic Areas” (24BMZ052).

## Supporting information

S1 Data (DOC)

S2 Data (DOC)
