# Supplementary material for: Analysis of the quadruple evolutionary game of ecosystem service payment empowered by farmers’ cooperatives
Source: PLoS One. 2025 Sep 4;20(9):e0329470. doi: 10.1371/journal.pone.0329470 (PMC12410733; doi:10.1371/journal.pone.0329470)
Supplement: S1 Data — (DOC) [file pone.0329470.s001.doc]

1. **graph**

function dydt=liyifenxiang(~,y,C1,C2,C3,C4,C5,C6,C7,Ca,Cb,S1,S2,A,E1,E2,E3,E4,E5,E6,Em,En,Ea,~,Ec,Ep,B,F,R,r,U1,U2)

dydt=zeros(4,1);

dydt(1)=y(1)*(1-y(1))*(-C1-y(3)*S2+y(3)*A-y(2)*S1+y(2)*A+Ea+Ec);

dydt(2)=y(2)*(1-y(2))*(y(3)*E5-y(3)*C6+y(1)*S1-y(1)*A-C2+E1+C3+Ep-E2);

dydt(3)=y(3)*(1-y(3))*(y(2)*E6-y(2)*C7+y(1)*S2-y(1)*A-C4+E3+R-Ca-Cb+Em+En+C5-E4);

dydt(4)=y(4)*(1-y(4))*(y(2)*y(3)*F+y(3)*U1+y(3)*B+r*U2-2*y(3)*r*U2-U2+y(3)*U2);

end

**Figure 5**

%% x variation

clc;clear;figure(1);

C1=1;C2=8.7;C3=5.8;C4=3.2;C5=2;C6=55;C7=60;Ca=0.3;Cb=0.8;S1=30;S2=32;A=35;E1=11;E2=8;E3=9;E4=6;E5=1.2;E6=1.6;Em=2.2;En=1.3;Ea=2;Eb=1.5;Ec=0.8;Ep=1;B=0.8;F=0.9;R=2.3;r=0.5;U1=5.4;U2=3.6;

[t,y]=ode45(@(t,y) liyifenxiang(t,y,C1,C2,C3,C4,C5,C6,C7,Ca,Cb,S1,S2,A,E1,E2,E3,E4,E5,E6,Em,En,Ea,Eb,Ec,Ep,B,F,R,r,U1,U2),[0,200],[0.3,0.5,0.5,0.5]);

plot3(y(:,1),y(:,2),y(:,4),'b-','linewidth',1);hold on

[t,y]=ode45(@(t,y) liyifenxiang(t,y,C1,C2,C3,C4,C5,C6,C7,Ca,Cb,S1,S2,A,E1,E2,E3,E4,E5,E6,Em,En,Ea,Eb,Ec,Ep,B,F,R,r,U1,U2),[0,200],[0.5,0.5,0.5,0.5]);

plot3(y(:,1),y(:,2),y(:,4),'r-.','linewidth',1);hold on

[t,y]=ode45(@(t,y) liyifenxiang(t,y,C1,C2,C3,C4,C5,C6,C7,Ca,Cb,S1,S2,A,E1,E2,E3,E4,E5,E6,Em,En,Ea,Eb,Ec,Ep,B,F,R,r,U1,U2),[0,200],[0.7,0.5,0.5,0.5]);

plot3(y(:,1),y(:,2),y(:,4),'g--','linewidth',1);hold on

[t,y]=ode45(@(t,y) liyifenxiang(t,y,C1,C2,C3,C4,C5,C6,C7,Ca,Cb,S1,S2,A,E1,E2,E3,E4,E5,E6,Em,En,Ea,Eb,Ec,Ep,B,F,R,r,U1,U2),[0,200],[0.9,0.5,0.5,0.5]);

plot3(y(:,1),y(:,2),y(:,4),'kp-','linewidth',1);hold on;grid on;

axis([0 1 0 1 0 1]);

xlabel('z');ylabel('y');zlabel('m');

legend('x=0.3','x=0.5','x=0.7','x=0.9');

%% y variation

clc;clear;figure(2);

C1=1;C2=8.7;C3=5.8;C4=3.2;C5=2;C6=55;C7=60;Ca=0.3;Cb=0.8;S1=30;S2=32;A=35;E1=11;E2=8;E3=9;E4=6;E5=1.2;E6=1.6;Em=2.2;En=1.3;Ea=2;Eb=1.5;Ec=0.8;Ep=1;B=0.8;F=0.9;R=2.3;r=0.5;U1=5.4;U2=3.6;

[t,y]=ode45(@(t,y) liyifenxiang(t,y,C1,C2,C3,C4,C5,C6,C7,Ca,Cb,S1,S2,A,E1,E2,E3,E4,E5,E6,Em,En,Ea,Eb,Ec,Ep,B,F,R,r,U1,U2),[0,200],[0.5,0.3,0.5,0.5]);

plot3(y(:,1),y(:,2),y(:,4),'b-','linewidth',1);hold on

[t,y]=ode45(@(t,y) liyifenxiang(t,y,C1,C2,C3,C4,C5,C6,C7,Ca,Cb,S1,S2,A,E1,E2,E3,E4,E5,E6,Em,En,Ea,Eb,Ec,Ep,B,F,R,r,U1,U2),[0,200],[0.5,0.5,0.5,0.5]);

plot3(y(:,1),y(:,2),y(:,4),'r-.','linewidth',1);hold on

[t,y]=ode45(@(t,y) liyifenxiang(t,y,C1,C2,C3,C4,C5,C6,C7,Ca,Cb,S1,S2,A,E1,E2,E3,E4,E5,E6,Em,En,Ea,Eb,Ec,Ep,B,F,R,r,U1,U2),[0,200],[0.5,0.7,0.5,0.5]);

plot3(y(:,1),y(:,2),y(:,4),'g--','linewidth',1);hold on

[t,y]=ode45(@(t,y) liyifenxiang(t,y,C1,C2,C3,C4,C5,C6,C7,Ca,Cb,S1,S2,A,E1,E2,E3,E4,E5,E6,Em,En,Ea,Eb,Ec,Ep,B,F,R,r,U1,U2),[0,200],[0.5,0.9,0.5,0.5]);

plot3(y(:,1),y(:,2),y(:,4),'kp-','linewidth',1);hold on;grid on;

axis([0 1 0 1 0 1]);

xlabel('z');ylabel('y');zlabel('m');

legend('y=0.3','y=0.5','y=0.7','y=0.9');

%% z variation

clc;clear;figure(3);

C1=1;C2=8.7;C3=5.8;C4=3.2;C5=2;C6=55;C7=60;Ca=0.3;Cb=0.8;S1=30;S2=32;A=35;E1=11;E2=8;E3=9;E4=6;E5=1.2;E6=1.6;Em=2.2;En=1.3;Ea=2;Eb=1.5;Ec=0.8;Ep=1;B=0.8;F=0.9;R=2.3;r=0.5;U1=5.4;U2=3.6;

[t,y]=ode45(@(t,y) liyifenxiang(t,y,C1,C2,C3,C4,C5,C6,C7,Ca,Cb,S1,S2,A,E1,E2,E3,E4,E5,E6,Em,En,Ea,Eb,Ec,Ep,B,F,R,r,U1,U2),[0,200],[0.5,0.5,0.3,0.5]);

plot3(y(:,1),y(:,2),y(:,4),'b-','linewidth',1);hold on

[t,y]=ode45(@(t,y) liyifenxiang(t,y,C1,C2,C3,C4,C5,C6,C7,Ca,Cb,S1,S2,A,E1,E2,E3,E4,E5,E6,Em,En,Ea,Eb,Ec,Ep,B,F,R,r,U1,U2),[0,200],[0.5,0.5,0.5,0.5]);

plot3(y(:,1),y(:,2),y(:,4),'r-.','linewidth',1);hold on

[t,y]=ode45(@(t,y) liyifenxiang(t,y,C1,C2,C3,C4,C5,C6,C7,Ca,Cb,S1,S2,A,E1,E2,E3,E4,E5,E6,Em,En,Ea,Eb,Ec,Ep,B,F,R,r,U1,U2),[0,200],[0.5,0.5,0.7,0.5]);

plot3(y(:,1),y(:,2),y(:,4),'g--','linewidth',1);hold on

[t,y]=ode45(@(t,y) liyifenxiang(t,y,C1,C2,C3,C4,C5,C6,C7,Ca,Cb,S1,S2,A,E1,E2,E3,E4,E5,E6,Em,En,Ea,Eb,Ec,Ep,B,F,R,r,U1,U2),[0,200],[0.5,0.5,0.9,0.5]);

plot3(y(:,1),y(:,2),y(:,4),'kp-','linewidth',1);hold on;grid on;

axis([0 1 0 1 0 1]);

xlabel('z');ylabel('y');zlabel('m');

legend('z=0.3','z=0.5','z=0.7','z=0.9');

%% m variation

clc;clear;figure(4);

C1=1;C2=8.7;C3=5.8;C4=3.2;C5=2;C6=55;C7=60;Ca=0.3;Cb=0.8;S1=30;S2=32;A=35;E1=11;E2=8;E3=9;E4=6;E5=1.2;E6=1.6;Em=2.2;En=1.3;Ea=2;Eb=1.5;Ec=0.8;Ep=1;B=0.8;F=0.9;R=2.3;r=0.5;U1=5.4;U2=3.6;

[t,y]=ode45(@(t,y) liyifenxiang(t,y,C1,C2,C3,C4,C5,C6,C7,Ca,Cb,S1,S2,A,E1,E2,E3,E4,E5,E6,Em,En,Ea,Eb,Ec,Ep,B,F,R,r,U1,U2),[0,200],[0.5,0.5,0.5,0.3]);

plot3(y(:,1),y(:,2),y(:,4),'b-','linewidth',1);hold on

[t,y]=ode45(@(t,y) liyifenxiang(t,y,C1,C2,C3,C4,C5,C6,C7,Ca,Cb,S1,S2,A,E1,E2,E3,E4,E5,E6,Em,En,Ea,Eb,Ec,Ep,B,F,R,r,U1,U2),[0,200],[0.5,0.5,0.5,0.5]);

plot3(y(:,1),y(:,2),y(:,4),'r-.','linewidth',1);hold on

[t,y]=ode45(@(t,y) liyifenxiang(t,y,C1,C2,C3,C4,C5,C6,C7,Ca,Cb,S1,S2,A,E1,E2,E3,E4,E5,E6,Em,En,Ea,Eb,Ec,Ep,B,F,R,r,U1,U2),[0,200],[0.5,0.5,0.5,0.7]);

plot3(y(:,1),y(:,2),y(:,4),'g--','linewidth',1);hold on

[t,y]=ode45(@(t,y) liyifenxiang(t,y,C1,C2,C3,C4,C5,C6,C7,Ca,Cb,S1,S2,A,E1,E2,E3,E4,E5,E6,Em,En,Ea,Eb,Ec,Ep,B,F,R,r,U1,U2),[0,200],[0.5,0.5,0.5,0.9]);

plot3(y(:,1),y(:,2),y(:,4),'kp-','linewidth',1);hold on;grid on;

axis([0 1 0 1 0 1]);

xlabel('z');ylabel('y');zlabel('m');

legend('m=0.3','m=0.5','m=0.7','m=0.9');

**Figure 6**

%% z=0

clc;clear;figure(7);

C1=1;C2=8.7;C3=5.8;C4=3.2;C5=2;C6=55;C7=60;Ca=0.3;Cb=0.8;S1=30;S2=32;A=35;E1=11;E2=8;E3=9;E4=6;E5=1.2;E6=1.6;Em=2.2;En=1.3;Ea=2;Eb=1.5;Ec=0.8;Ep=1;B=0.8;F=0.9;R=2.3;r=0.5;U1=5.4;U2=3.6;

[t,y]=ode45(@(t,y) liyifenxiang(t,y,C1,C2,C3,C4,C5,C6,C7,Ca,Cb,S1,S2,A,E1,E2,E3,E4,E5,E6,Em,En,Ea,Eb,Ec,Ep,B,F,R,r,U1,U2),[0,200],[0.5,0.5,0,0.5]);

plot3(y(:,1),y(:,2),y(:,4),'r:+','linewidth',1);hold on

C1=1;C2=8.7;C3=5.8;C4=3.2;C5=2;C6=55;C7=60;Ca=0.3;Cb=0.8;S1=30;S2=32;A=40;E1=11;E2=8;E3=9;E4=6;E5=1.2;E6=1.6;Em=2.2;En=1.3;Ea=2;Eb=1.5;Ec=0.8;Ep=1;B=0.8;F=0.9;R=2.3;r=0.5;U1=5.4;U2=3.6;

[t,y]=ode45(@(t,y) liyifenxiang(t,y,C1,C2,C3,C4,C5,C6,C7,Ca,Cb,S1,S2,A,E1,E2,E3,E4,E5,E6,Em,En,Ea,Eb,Ec,Ep,B,F,R,r,U1,U2),[0,200],[0.5,0.5,0,0.5]);

plot3(y(:,1),y(:,2),y(:,4),'b-o','linewidth',1);hold on

C1=1;C2=8.7;C3=5.8;C4=3.2;C5=2;C6=55;C7=60;Ca=0.3;Cb=0.8;S1=30;S2=32;A=45;E1=11;E2=8;E3=9;E4=6;E5=1.2;E6=1.6;Em=2.2;En=1.3;Ea=2;Eb=1.5;Ec=0.8;Ep=1;B=0.8;F=0.9;R=2.3;r=0.5;U1=5.4;U2=3.6;

[t,y]=ode45(@(t,y) liyifenxiang(t,y,C1,C2,C3,C4,C5,C6,C7,Ca,Cb,S1,S2,A,E1,E2,E3,E4,E5,E6,Em,En,Ea,Eb,Ec,Ep,B,F,R,r,U1,U2),[0,200],[0.5,0.5,0,0.5]);

plot3(y(:,1),y(:,2),y(:,4),'k-.','linewidth',1);hold on

C1=1;C2=8.7;C3=5.8;C4=3.2;C5=2;C6=55;C7=60;Ca=0.3;Cb=0.8;S1=30;S2=32;A=50;E1=11;E2=8;E3=9;E4=6;E5=1.2;E6=1.6;Em=2.2;En=1.3;Ea=2;Eb=1.5;Ec=0.8;Ep=1;B=0.8;F=0.9;R=2.3;r=0.5;U1=5.4;U2=3.6;

[t,y]=ode45(@(t,y) liyifenxiang(t,y,C1,C2,C3,C4,C5,C6,C7,Ca,Cb,S1,S2,A,E1,E2,E3,E4,E5,E6,Em,En,Ea,Eb,Ec,Ep,B,F,R,r,U1,U2),[0,200],[0.5,0.5,0,0.5]);

plot3(y(:,1),y(:,2),y(:,4),'g--','linewidth',1);hold on

axis([0 1 0 1 0 1]);

xlabel('x');ylabel('y');zlabel('m');

legend('A=35','A=40','A=45','A=50');

grid on

hold on

set(0,'defaultfigurecolor','w')

%% z=0.5

clc;clear;figure(8);

C1=1;C2=8.7;C3=5.8;C4=3.2;C5=2;C6=55;C7=60;Ca=0.3;Cb=0.8;S1=30;S2=32;A=35;E1=11;E2=8;E3=9;E4=6;E5=1.2;E6=1.6;Em=2.2;En=1.3;Ea=2;Eb=1.5;Ec=0.8;Ep=1;B=0.8;F=0.9;R=2.3;r=0.5;U1=5.4;U2=3.6;

[t,y]=ode45(@(t,y) liyifenxiang(t,y,C1,C2,C3,C4,C5,C6,C7,Ca,Cb,S1,S2,A,E1,E2,E3,E4,E5,E6,Em,En,Ea,Eb,Ec,Ep,B,F,R,r,U1,U2),[0,200],[0.5,0.5,0.5,0.5]);

plot3(y(:,1),y(:,2),y(:,4),'r:+','linewidth',1);hold on

C1=1;C2=8.7;C3=5.8;C4=3.2;C5=2;C6=55;C7=60;Ca=0.3;Cb=0.8;S1=30;S2=32;A=40;E1=11;E2=8;E3=9;E4=6;E5=1.2;E6=1.6;Em=2.2;En=1.3;Ea=2;Eb=1.5;Ec=0.8;Ep=1;B=0.8;F=0.9;R=2.3;r=0.5;U1=5.4;U2=3.6;

[t,y]=ode45(@(t,y) liyifenxiang(t,y,C1,C2,C3,C4,C5,C6,C7,Ca,Cb,S1,S2,A,E1,E2,E3,E4,E5,E6,Em,En,Ea,Eb,Ec,Ep,B,F,R,r,U1,U2),[0,200],[0.5,0.5,0.5,0.5]);

plot3(y(:,1),y(:,2),y(:,4),'b-o','linewidth',1);hold on

C1=1;C2=8.7;C3=5.8;C4=3.2;C5=2;C6=55;C7=60;Ca=0.3;Cb=0.8;S1=30;S2=32;A=45;E1=11;E2=8;E3=9;E4=6;E5=1.2;E6=1.6;Em=2.2;En=1.3;Ea=2;Eb=1.5;Ec=0.8;Ep=1;B=0.8;F=0.9;R=2.3;r=0.5;U1=5.4;U2=3.6;

[t,y]=ode45(@(t,y) liyifenxiang(t,y,C1,C2,C3,C4,C5,C6,C7,Ca,Cb,S1,S2,A,E1,E2,E3,E4,E5,E6,Em,En,Ea,Eb,Ec,Ep,B,F,R,r,U1,U2),[0,200],[0.5,0.5,0.5,0.5]);

plot3(y(:,1),y(:,2),y(:,4),'k-.','linewidth',1);hold on

C1=1;C2=8.7;C3=5.8;C4=3.2;C5=2;C6=55;C7=60;Ca=0.3;Cb=0.8;S1=30;S2=32;A=50;E1=11;E2=8;E3=9;E4=6;E5=1.2;E6=1.6;Em=2.2;En=1.3;Ea=2;Eb=1.5;Ec=0.8;Ep=1;B=0.8;F=0.9;R=2.3;r=0.5;U1=5.4;U2=3.6;

[t,y]=ode45(@(t,y) liyifenxiang(t,y,C1,C2,C3,C4,C5,C6,C7,Ca,Cb,S1,S2,A,E1,E2,E3,E4,E5,E6,Em,En,Ea,Eb,Ec,Ep,B,F,R,r,U1,U2),[0,200],[0.5,0.5,0.5,0.5]);

plot3(y(:,1),y(:,2),y(:,4),'g--','linewidth',1);hold on

axis([0 1 0 1 0 1]);

xlabel('x');ylabel('y');zlabel('m');

legend('A=35','A=40','A=45','A=50');

grid on

hold on

set(0,'defaultfigurecolor','w')

%% z=1

clc;clear;figure(10);

C1=1;C2=8.7;C3=5.8;C4=3.2;C5=2;C6=55;C7=60;Ca=0.3;Cb=0.8;S1=30;S2=32;A=35;E1=11;E2=8;E3=9;E4=6;E5=1.2;E6=1.6;Em=2.2;En=1.3;Ea=2;Eb=1.5;Ec=0.8;Ep=1;B=0.8;F=0.9;R=2.3;r=0.5;U1=5.4;U2=3.6;

[t,y]=ode45(@(t,y) liyifenxiang(t,y,C1,C2,C3,C4,C5,C6,C7,Ca,Cb,S1,S2,A,E1,E2,E3,E4,E5,E6,Em,En,Ea,Eb,Ec,Ep,B,F,R,r,U1,U2),[0,200],[0.5,0.5,1,0.5]);

plot3(y(:,1),y(:,2),y(:,4),'r:+','linewidth',1);hold on

C1=1;C2=8.7;C3=5.8;C4=3.2;C5=2;C6=55;C7=60;Ca=0.3;Cb=0.8;S1=30;S2=32;A=40;E1=11;E2=8;E3=9;E4=6;E5=1.2;E6=1.6;Em=2.2;En=1.3;Ea=2;Eb=1.5;Ec=0.8;Ep=1;B=0.8;F=0.9;R=2.3;r=0.5;U1=5.4;U2=3.6;

[t,y]=ode45(@(t,y) liyifenxiang(t,y,C1,C2,C3,C4,C5,C6,C7,Ca,Cb,S1,S2,A,E1,E2,E3,E4,E5,E6,Em,En,Ea,Eb,Ec,Ep,B,F,R,r,U1,U2),[0,200],[0.5,0.5,1,0.5]);

plot3(y(:,1),y(:,2),y(:,4),'b-o','linewidth',1);hold on

C1=1;C2=8.7;C3=5.8;C4=3.2;C5=2;C6=55;C7=60;Ca=0.3;Cb=0.8;S1=30;S2=32;A=45;E1=11;E2=8;E3=9;E4=6;E5=1.2;E6=1.6;Em=2.2;En=1.3;Ea=2;Eb=1.5;Ec=0.8;Ep=1;B=0.8;F=0.9;R=2.3;r=0.5;U1=5.4;U2=3.6;

[t,y]=ode45(@(t,y) liyifenxiang(t,y,C1,C2,C3,C4,C5,C6,C7,Ca,Cb,S1,S2,A,E1,E2,E3,E4,E5,E6,Em,En,Ea,Eb,Ec,Ep,B,F,R,r,U1,U2),[0,200],[0.5,0.5,1,0.5]);

plot3(y(:,1),y(:,2),y(:,4),'k-.','linewidth',1);hold on

C1=1;C2=8.7;C3=5.8;C4=3.2;C5=2;C6=55;C7=60;Ca=0.3;Cb=0.8;S1=30;S2=32;A=50;E1=11;E2=8;E3=9;E4=6;E5=1.2;E6=1.6;Em=2.2;En=1.3;Ea=2;Eb=1.5;Ec=0.8;Ep=1;B=0.8;F=0.9;R=2.3;r=0.5;U1=5.4;U2=3.6;

[t,y]=ode45(@(t,y) liyifenxiang(t,y,C1,C2,C3,C4,C5,C6,C7,Ca,Cb,S1,S2,A,E1,E2,E3,E4,E5,E6,Em,En,Ea,Eb,Ec,Ep,B,F,R,r,U1,U2),[0,200],[0.5,0.5,1,0.5]);

plot3(y(:,1),y(:,2),y(:,4),'g--','linewidth',1);hold on

axis([0 1 0 1 0 1]);

xlabel('x');ylabel('y');zlabel('m');

legend('A=35','A=40','A=45','A=50');

grid on

hold on

set(0,'defaultfigurecolor','w')

**Figure 7**

%% x=0

clc;clear;figure(7);

C1=1;C2=8.7;C3=5.8;C4=3.2;C5=2;C6=55;C7=60;Ca=0.3;Cb=0.8;S1=30;S2=32;A=35;E1=11;E2=8;E3=9;E4=6;E5=1.2;E6=1.6;Em=2.2;En=1.3;Ea=2;Eb=1.5;Ec=0.8;Ep=1;B=0.8;F=0.9;R=2.3;r=0.5;U1=5.4;U2=3.6;

[t,y]=ode45(@(t,y) liyifenxiang(t,y,C1,C2,C3,C4,C5,C6,C7,Ca,Cb,S1,S2,A,E1,E2,E3,E4,E5,E6,Em,En,Ea,Eb,Ec,Ep,B,F,R,r,U1,U2),[0,200],[0.5,0.5,0,0.5]);

plot3(y(:,1),y(:,2),y(:,4),'r:+','linewidth',1);hold on

C1=1;C2=8.7;C3=5.8;C4=3.2;C5=2;C6=55;C7=60;Ca=0.3;Cb=0.8;S1=30;S2=42;A=35;E1=11;E2=8;E3=9;E4=20;E5=1.2;E6=1.6;Em=2.2;En=1.3;Ea=2;Eb=1.5;Ec=0.8;Ep=1;B=0.8;F=0.9;R=2.3;r=0.5;U1=5.4;U2=3.6;

[t,y]=ode45(@(t,y) liyifenxiang(t,y,C1,C2,C3,C4,C5,C6,C7,Ca,Cb,S1,S2,A,E1,E2,E3,E4,E5,E6,Em,En,Ea,Eb,Ec,Ep,B,F,R,r,U1,U2),[0,200],[0.5,0.5,0,0.5]);

plot3(y(:,1),y(:,2),y(:,4),'b-o','linewidth',1);hold on

C1=1;C2=8.7;C3=5.8;C4=3.2;C5=2;C6=55;C7=60;Ca=0.3;Cb=0.8;S1=30;S2=52;A=35;E1=11;E2=8;E3=9;E4=40;E5=1.2;E6=1.6;Em=2.2;En=1.3;Ea=2;Eb=1.5;Ec=0.8;Ep=1;B=0.8;F=0.9;R=2.3;r=0.5;U1=5.4;U2=3.6;

[t,y]=ode45(@(t,y) liyifenxiang(t,y,C1,C2,C3,C4,C5,C6,C7,Ca,Cb,S1,S2,A,E1,E2,E3,E4,E5,E6,Em,En,Ea,Eb,Ec,Ep,B,F,R,r,U1,U2),[0,200],[0.5,0.5,0,0.5]);

plot3(y(:,1),y(:,2),y(:,4),'k-.','linewidth',1);hold on

C1=1;C2=8.7;C3=5.8;C4=3.2;C5=2;C6=55;C7=60;Ca=0.3;Cb=0.8;S1=30;S2=62;A=35;E1=11;E2=8;E3=9;E4=60;E5=1.2;E6=1.6;Em=2.2;En=1.3;Ea=2;Eb=1.5;Ec=0.8;Ep=1;B=0.8;F=0.9;R=2.3;r=0.5;U1=5.4;U2=3.6;

[t,y]=ode45(@(t,y) liyifenxiang(t,y,C1,C2,C3,C4,C5,C6,C7,Ca,Cb,S1,S2,A,E1,E2,E3,E4,E5,E6,Em,En,Ea,Eb,Ec,Ep,B,F,R,r,U1,U2),[0,200],[0.5,0.5,0,0.5]);

plot3(y(:,1),y(:,2),y(:,4),'g--','linewidth',1);hold on

axis([0 1 0 1 0 1]);

xlabel('x');ylabel('y');zlabel('m');

legend('S2=32','S2=42','S2=52','S2=62');

grid on

hold on

set(0,'defaultfigurecolor','w')

%% x=0.5

clc;clear;figure(7);

C1=1;C2=8.7;C3=5.8;C4=3.2;C5=2;C6=55;C7=60;Ca=0.3;Cb=0.8;S1=30;S2=32;A=35;E1=11;E2=8;E3=9;E4=6;E5=1.2;E6=1.6;Em=2.2;En=1.3;Ea=2;Eb=1.5;Ec=0.8;Ep=1;B=0.8;F=0.9;R=2.3;r=0.5;U1=5.4;U2=3.6;

[t,y]=ode45(@(t,y) liyifenxiang(t,y,C1,C2,C3,C4,C5,C6,C7,Ca,Cb,S1,S2,A,E1,E2,E3,E4,E5,E6,Em,En,Ea,Eb,Ec,Ep,B,F,R,r,U1,U2),[0,200],[0.5,0.5,0.5,0.5]);

plot3(y(:,1),y(:,2),y(:,4),'r:+','linewidth',1);hold on

C1=1;C2=8.7;C3=5.8;C4=3.2;C5=2;C6=55;C7=60;Ca=0.3;Cb=0.8;S1=30;S2=42;A=35;E1=11;E2=8;E3=9;E4=20;E5=1.2;E6=1.6;Em=2.2;En=1.3;Ea=2;Eb=1.5;Ec=0.8;Ep=1;B=0.8;F=0.9;R=2.3;r=0.5;U1=5.4;U2=3.6;

[t,y]=ode45(@(t,y) liyifenxiang(t,y,C1,C2,C3,C4,C5,C6,C7,Ca,Cb,S1,S2,A,E1,E2,E3,E4,E5,E6,Em,En,Ea,Eb,Ec,Ep,B,F,R,r,U1,U2),[0,200],[0.5,0.5,0.5,0.5]);

plot3(y(:,1),y(:,2),y(:,4),'b-o','linewidth',1);hold on

C1=1;C2=8.7;C3=5.8;C4=3.2;C5=2;C6=55;C7=60;Ca=0.3;Cb=0.8;S1=30;S2=52;A=35;E1=11;E2=8;E3=9;E4=40;E5=1.2;E6=1.6;Em=2.2;En=1.3;Ea=2;Eb=1.5;Ec=0.8;Ep=1;B=0.8;F=0.9;R=2.3;r=0.5;U1=5.4;U2=3.6;

[t,y]=ode45(@(t,y) liyifenxiang(t,y,C1,C2,C3,C4,C5,C6,C7,Ca,Cb,S1,S2,A,E1,E2,E3,E4,E5,E6,Em,En,Ea,Eb,Ec,Ep,B,F,R,r,U1,U2),[0,200],[0.5,0.5,0.5,0.5]);

plot3(y(:,1),y(:,2),y(:,4),'k-.','linewidth',1);hold on

C1=1;C2=8.7;C3=5.8;C4=3.2;C5=2;C6=55;C7=60;Ca=0.3;Cb=0.8;S1=30;S2=62;A=35;E1=11;E2=8;E3=9;E4=60;E5=1.2;E6=1.6;Em=2.2;En=1.3;Ea=2;Eb=1.5;Ec=0.8;Ep=1;B=0.8;F=0.9;R=2.3;r=0.5;U1=5.4;U2=3.6;

[t,y]=ode45(@(t,y) liyifenxiang(t,y,C1,C2,C3,C4,C5,C6,C7,Ca,Cb,S1,S2,A,E1,E2,E3,E4,E5,E6,Em,En,Ea,Eb,Ec,Ep,B,F,R,r,U1,U2),[0,200],[0.5,0.5,0.5,0.5]);

plot3(y(:,1),y(:,2),y(:,4),'g--','linewidth',1);hold on

axis([0 1 0 1 0 1]);

xlabel('x');ylabel('y');zlabel('m');

legend('S2=32','S2=42','S2=52','S2=62');

grid on

hold on

set(0,'defaultfigurecolor','w')

%% x=1

clc;clear;figure(7);

C1=1;C2=8.7;C3=5.8;C4=3.2;C5=2;C6=55;C7=60;Ca=0.3;Cb=0.8;S1=30;S2=32;A=35;E1=11;E2=8;E3=9;E4=6;E5=1.2;E6=1.6;Em=2.2;En=1.3;Ea=2;Eb=1.5;Ec=0.8;Ep=1;B=0.8;F=0.9;R=2.3;r=0.5;U1=5.4;U2=3.6;

[t,y]=ode45(@(t,y) liyifenxiang(t,y,C1,C2,C3,C4,C5,C6,C7,Ca,Cb,S1,S2,A,E1,E2,E3,E4,E5,E6,Em,En,Ea,Eb,Ec,Ep,B,F,R,r,U1,U2),[0,200],[0.5,0.5,1,0.5]);

plot3(y(:,1),y(:,2),y(:,4),'r:+','linewidth',1);hold on

C1=1;C2=8.7;C3=5.8;C4=3.2;C5=2;C6=55;C7=60;Ca=0.3;Cb=0.8;S1=30;S2=42;A=35;E1=11;E2=8;E3=9;E4=20;E5=1.2;E6=1.6;Em=2.2;En=1.3;Ea=2;Eb=1.5;Ec=0.8;Ep=1;B=0.8;F=0.9;R=2.3;r=0.5;U1=5.4;U2=3.6;

[t,y]=ode45(@(t,y) liyifenxiang(t,y,C1,C2,C3,C4,C5,C6,C7,Ca,Cb,S1,S2,A,E1,E2,E3,E4,E5,E6,Em,En,Ea,Eb,Ec,Ep,B,F,R,r,U1,U2),[0,200],[0.5,0.5,1,0.5]);

plot3(y(:,1),y(:,2),y(:,4),'b-o','linewidth',1);hold on

C1=1;C2=8.7;C3=5.8;C4=3.2;C5=2;C6=55;C7=60;Ca=0.3;Cb=0.8;S1=30;S2=52;A=35;E1=11;E2=8;E3=9;E4=40;E5=1.2;E6=1.6;Em=2.2;En=1.3;Ea=2;Eb=1.5;Ec=0.8;Ep=1;B=0.8;F=0.9;R=2.3;r=0.5;U1=5.4;U2=3.6;

[t,y]=ode45(@(t,y) liyifenxiang(t,y,C1,C2,C3,C4,C5,C6,C7,Ca,Cb,S1,S2,A,E1,E2,E3,E4,E5,E6,Em,En,Ea,Eb,Ec,Ep,B,F,R,r,U1,U2),[0,200],[0.5,0.5,1,0.5]);

plot3(y(:,1),y(:,2),y(:,4),'k-.','linewidth',1);hold on

C1=1;C2=8.7;C3=5.8;C4=3.2;C5=2;C6=55;C7=60;Ca=0.3;Cb=0.8;S1=30;S2=62;A=35;E1=11;E2=8;E3=9;E4=60;E5=1.2;E6=1.6;Em=2.2;En=1.3;Ea=2;Eb=1.5;Ec=0.8;Ep=1;B=0.8;F=0.9;R=2.3;r=0.5;U1=5.4;U2=3.6;

[t,y]=ode45(@(t,y) liyifenxiang(t,y,C1,C2,C3,C4,C5,C6,C7,Ca,Cb,S1,S2,A,E1,E2,E3,E4,E5,E6,Em,En,Ea,Eb,Ec,Ep,B,F,R,r,U1,U2),[0,200],[0.5,0.5,1,0.5]);

plot3(y(:,1),y(:,2),y(:,4),'g--','linewidth',1);hold on

axis([0 1 0 1 0 1]);

xlabel('x');ylabel('y');zlabel('m');

legend('S2=32','S2=42','S2=52','S2=62');

grid on

hold on

set(0,'defaultfigurecolor','w')

1. **Calculation process**
2. **Local Government Incentives** (Incentive)、Enterprise Ecological Product Management、Cooperative Ecological Product Production、Members Participate in Ecological Product Production

a_1_:-C_1_-S_1_-S_2_+2A+E_a_+E_b_

b_1_:-C_2_-C_6_-A+S_1_+E_1_+E_p_+E_5_

c_1_:-C_4_-C_7_-A+S_2_+E_3_+R+E_6_-C_a_-C_b_+E_m_+E_n_

d_1_:U_1_+B+F

1. **Local Government Incentives** (Incentive)、Enterprise Ecological Product Management、Cooperative Ecological Product Production、Members Participate in Traditional Product Production

a_2_:-C_1_-S_1_-S_2_+2A+E_a_+E_b_

b_2_:-C_2_-C_6_-A+S_1_+E_1_+E_p_+E_5_

c_2_:-C_4_-C_7_-A+S_2_+E_3_+R+E_6_-C_a_-C_b_+E_m_+E_n_

d_2_:rU_2_

1. **Local Government Incentives** (Incentive)、Enterprise Ecological Product Management、Cooperative Traditional Product Production、Members Participate in Ecological Product Production

a_3_:-C_1_-S_1_+A+E_a_+E_b_

b_3_:-C_2_-A+S_1_+E_1_+E_p_

c_3_:-C_5_+E_4_

d_3_:rU_2_

1. **Local Government Incentives** (Incentive)、Enterprise Ecological Product Management、Cooperative Traditional Product Production、Members Participate in Traditional Product Production

a_4_:-C_1_-S_1_+A+E_a_+E_b_

b_4_:-C_2_-A+S_1_+E_1_+E_p_

c_4_:-C_5_+E_4_

d_4_:U_2_

1. **Local Government Incentives** (Incentive)、Enterprise Traditional Product Management、Cooperative Ecological Product Production、Members Participate in Ecological Product Production

a_5_:-C_1_-S_2_+A+E_a_+E_b_

b_5_:-C_3_+E_2_

c_5_:-C_4_-A+S_2_+E_3_+R-C_a_-C_b_+E_m_+E_n_

d_5_:U_1_+B

1. **Local Government Incentives** (Incentive)、Enterprise Traditional Product Management、Cooperative Ecological Product Production、Members Participate in Traditional Product Production

a_6_:-C_1_-S_2_+A+E_a_+E_b_

b_6_:-C_3_+E_2_

c_6_:-C_4_-A+S_2_+E_3_+R-C_a_-C_b_+E_m_+E_n_

d_6_:rU_2_

1. **Local Government Incentives** (Incentive)、Enterprise Traditional Product Management、Cooperative Traditional Product Production、Members Participate in Ecological Product Production

a_7_:-C_1_+E_a_

b_7_:-C_3_+E_2_

c_7_:-C_5_+E_4_

D_7_:rU_2_

1. **Local Government Incentives** (Incentive)、Enterprise Traditional Product Management、Cooperative Traditional Product Production、Members Participate in Traditional Product Production

a_8_:-C_1_+E_a_

b_8_:-C_3_+E_2_

c_8_:-C_5_+E_4_

d_8_:U_2_

1. **Local Government Incentives** (Incentive)、Enterprise Ecological Product Management、Cooperative Ecological Product Production、Members Participate in Ecological Product Production

a_9_:E_b_-E_c_

b_9_:-C_2_-C_6_+E_1_+E_p_+E_5_

c_9_:-C_4_-C_7_+E_3_+R+E_6_-C_a_-C_b_+E_m_+E_n_

d_9_:U_1_+B+F

1. **Local Government Incentives** (Incentive)、Enterprise Ecological Product Management、Cooperative Ecological Product Production、Members Participate in Traditional Product Production

a_10_:E_b_-E_c_

b_10_:-C_2_-C_6_+E_1_+E_p_+E_5_

c_10_:-C_4_-C_7_+E_3_+R+E_6_-C_a_-C_b_+E_m_+E_n_

d_10_:rU_2_

1. **Local Government Incentives** (Incentive)、Enterprise Ecological Product Management、Cooperative Traditional Product Production、Members Participate in Ecological Product Production

a_11_:E_b_-E_c_

b_11_:-C_2_+E_1_+E_p_

c_11_:-C_5_+E_4_

d_11_:rU_2_

1. **Local Government Incentives** (Incentive)、Enterprise Ecological Product Management、Cooperative Traditional Product Production、Members Participate in Traditional Product Production

a_12_:E_b_-E_c_

b_12_:-C_2_+E_1_+E_p_

c_12_:-C_5_+E_4_

d_12_:U_2_

1. **Local Government Incentives** (Incentive)、Enterprise Traditional Product Management、Cooperative Ecological Product Production、Members Participate in Ecological Product Production

a_13_:E_b_-E_c_

b_13_:-C_3_+E_2_

c_13_:-C_4_+E_3_+R-C_a_-C_b_+E_m_+E_n_

d_13_:U_1_+B

1. **Local Government Incentives** (Incentive)、Enterprise Traditional Product Management、Cooperative Ecological Product Production、Members Participate in Traditional Product Production

a_14_:E_b_-E_c_

b_14_:-C_3_+E_2_

c_14_:-C_4_+E_3_+R-C_a_-C_b_+E_m_+E_n_

d_14_:rU_2_

1. **Local Government Incentives** (Incentive)、Enterprise Traditional Product Management、Cooperative Traditional Product Production、Members Participate in Ecological Product Production

a_15_:-E_c_

b_15_:-C_3_+E_2_

c_15_:-C_5_+E_4_

d_15_:rU_2_

1. **Local Government Incentives** (Incentive)、Enterprise Traditional Product Management、Cooperative Traditional Product Production、Members Participate in Traditional Product Production

a_16_:-E_c_

c_16_:-C_3_+E_2_

c_16_:-C_5_+E_4_

d_16_:U_2_

**3.Parameter setting**

| Parameter | C1 | C_2_ | C_3_ | C_4_ | C_5_ | C_6_ | C_7_ | C _a_ | C_b_ | S_1_ | S_2_ | A | E_1_ | E_2_ | E_3_ |
| --- | --- | --- | --- | --- | --- | --- | --- | --- | --- | --- | --- | --- | --- | --- | --- |
| Assigned Value | 1 | 8.7 | 5.8 | 3.2 | 2 | 55 | 60 | 0.3 | 0.8 | 30 | 35 | 0.64 | 11 | 8 | 9 |
| Parameter | E_4_ | E_5_ | E_6_ | E_m_ | E_n_ | E_p_ | E_a_ | E_b_ | E_c_ | F | R | B | r | U_1_ | U_2_ |
| Assigned Value | 6 | 1.2 | 1.6 | 2.2 | 1.3 | 1 | 2 | 1.5 | 0.8 | 0.9 | 2.3 | 0.8 | 0.5 | 5.4 | 3.6 |
