# Supplementary material for: Analysis of the quadruple evolutionary game of ecosystem service payment empowered by farmers’ cooperatives
Source: PLoS One. 2025 Sep 4;20(9):e0329470. doi: 10.1371/journal.pone.0329470 (PMC12410733; doi:10.1371/journal.pone.0329470)
Supplement: S2 Data — (DOC) [file pone.0329470.s002.doc]

| Parameter | C1 | C_2_ | C_3_ | C_4_ | C_5_ | C_6_ | C_7_ | C _a_ | C_b_ | S_1_ | S_2_ | A | E_1_ | E_2_ | E_3_ |
| --- | --- | --- | --- | --- | --- | --- | --- | --- | --- | --- | --- | --- | --- | --- | --- |
| Assigned Value | 1 | 8.7 | 5.8 | 3.2 | 2 | 55 | 60 | 0.3 | 0.8 | 30 | 35 | 0.64 | 11 | 8 | 9 |
| Parameter | E_4_ | E_5_ | E_6_ | E_m_ | E_n_ | E_p_ | E_a_ | E_b_ | E_c_ | F | R | B | r | U_1_ | U_2_ |
| Assigned Value | 6 | 1.2 | 1.6 | 2.2 | 1.3 | 1 | 2 | 1.5 | 0.8 | 0.9 | 2.3 | 0.8 | 0.5 | 5.4 | 3.6 |
